# The role of hexokinases in epigenetic regulation: altered hexokinase expression and chromatin stability in yeast

**DOI:** 10.1186/s13072-024-00551-9

**Published:** 2024-08-27

**Authors:** Srinivasu Karri, Quinn Dickinson, Jing Jia, Yi Yang, Haiyun Gan, Zhiquan Wang, Yibin Deng, Chuanhe Yu

**Affiliations:** 1 Hormel Institute, University of Minnesota, Austin, MN 55912 USA; 2 CAS Key Laboratory of Quantitative Engineering Biology, Guangdong Provincial Key Laboratory of Synthetic Genomics and Shenzhen Key Laboratory of Synthetic Genomics, Shenzhen Institute of Synthetic Biology, Shenzhen Institutes of Advanced Technology, Chinese Academy of Sciences, Shenzhen, 518055 China; 3 Division of Hematology, Department of Medicine, Mayo Clinic, Rochester, MN 55905 USA; 4 Department of Urology, Masonic Cancer Center, University of Minnesota Medical School, Minneapolis, MN USA

**Keywords:** Hexokinase, 2-deoxy-D-glucose, Histone chaperones, eSPAN, Chromatin replication, Yeast, Chromatin stability, Histone methylation, Histone acetylation

## Abstract

**Background:**

Human hexokinase 2 (HK2) plays an important role in regulating Warburg effect, which metabolizes glucose to lactate acid even in the presence of ample oxygen and provides intermediate metabolites to support cancer cell proliferation and tumor growth. *HK2* overexpression has been observed in various types of cancers and targeting *HK2*-driven Warburg effect has been suggested as a potential cancer therapeutic strategy. Given that epigenetic enzymes utilize metabolic intermediates as substrates or co-factors to carry out post-translational modification of histones and nucleic acids modifications in cells, we hypothesized that altering *HK2* expression could impact the epigenome and, consequently, chromatin stability in yeast. To test this hypothesis, we established genetic models with different yeast hexokinase 2 (*HXK2)* expression in *Saccharomyces cerevisiae* yeast cells and investigated the effect of *HXK2*-dependent metabolism on parental nucleosome transfer, a key DNA replication–coupled epigenetic inheritance process, and chromatin stability.

**Results:**

By comparing the growth of mutant yeast cells carrying single deletion of *hxk1Δ*, *hxk2Δ*, or double-loss of *hxk1Δ hxk2Δ* to wild-type cells, we firstly confirmed that *HXK2* is the dominant *HXK* in yeast cell growth. Surprisingly, manipulating *HXK2* expression in yeast, whether through overexpression or deletion, had only a marginal impact on parental nucleosome assembly, but a noticeable trend with decrease chromatin instability. However, targeting yeast cells with 2-deoxy-D-glucose (2-DG), a clinical glycolysis inhibitor that has been proposed as an anti-cancer treatment, significantly increased chromatin instability.

**Conclusion:**

Our findings suggest that in yeast cells lacking *HXK2*, alternative *HXK*s such as *HXK1* or glucokinase 1 (*GLK1*) play a role in supporting glycolysis at a level that adequately maintains epigenomic stability. While our study demonstrated an increase in epigenetic instability with 2-DG treatment, the observed effect seemed to occur dependent on non-glycolytic function of Hxk2. Thus, additional research is needed to identify the molecular mechanism through which 2-DG influences chromatin stability.

**Supplementary Information:**

The online version contains supplementary material available at 10.1186/s13072-024-00551-9.

## Background

Hexokinases (HKs or HXKs), the rate-limiting enzyme responsible for glucose metabolism [1], catalyzes the phosphorylation of glucose to glucose-6-phosphate (Glc-6-P). Glc-6-P subsequently enters glycolysis (triose phosphate pathway) or pentose phosphate pathway, serving as a vital substrate for adenosine triphosphate (ATP) production and the biosynthesis of various metabolites (amino acids, nucleic acids, and lipids). There are four HK isozymes (HK1-4) in humans [2]. And each has a different tissue and organ distribution, as well as distinct metabolic functions [3]. Expression of some *HKs* is associated with worse prognosis in several tumor types. For example, *HK1* plays an oncogenic role in bladder cancer [4], and *HK2* enhances live cancer stemness [5]. High *HK2* expression has been linked to a variety kind of cancer and is associated with poor overall survival in cancer patients [6–8]. For these reasons, targeting HKs has been suggested as a potential strategy for cancer therapy [9, 10]. In support of this approach, deleting *HK2* can decrease cancer cell proliferation without prominent side effects in animal models, suggesting *HK2* is a promising target for cancer therapy [7]. Highly glycolytic proliferating cells, such as cancer cells, rely on *HK2* expression to accelerate glucose metabolism, even under hypoxemia conditions. However, this hightened metabolic activity leads to the generation of metabolic products such as acetyl-coenzyme A (Acetyl-CoA), which serve as substrates for numerous epigenetic modulators, fulfilling both the energy needs of the cells and influencing chromatin modifications. Additionally, there is evidence suggesting that yeast Hxk2 translocate into the nucleus, potentially influencing gene expression, and cell differentiation [11, 12]. To learn more about the utility of targeting *HK*s, it is important to characterize the effects of modulating *HK* expression on eukaryotic cells.

In this study, we investigated how abnormal yeast Hexokinase 2 (*HXK2)* expression affects epigenetics and chromatin instability in a simple, genetically tractable model system for eukaryotic cell function: the budding yeast *Saccharomyces cerevisiae*. In this organism, three genes encode proteins that phosphorylate glucose to Glc-6-P: *HXK1*,* HXK2*, and the glucokinase gene *GLK1*. *HXK2* appears to play the main role in glucose phosphorylation in vivo [13, 14]. Therefore, we investigated the effects of *HXK2* deletion and overexpression on parental histone H3-H4 transfer, an essential step in epigenetic inheritance [15]. We hypothesized that the DNA-replication coupled epigenetic inheritance process is altered because DNA replication is an energy consuming process. We discovered that both kinds of modified *HXK2* expression seemed to lessen chromatin instability and had only a little impact on the parental histone H3-H4 distribution on replicating chromatin. On the other hand, yeast chromatin instability was elevated by 2-deoxy-D-glucose (2-DG), a clinical glycolysis inhibitor that has been used as a cancer treatment [14]. Although 2-DG interacts with multiple cellular pathways and has various biological effects [16–18], 2-DG most well-known mechanism is related to glycolysis. Once inside the cell, 2-DG is phosphorylated by *HXK*s to form 2-deoxy-d-glucose-6-phosphate (2-DG-6-P), which, unlike G-6-P, cannot be further metabolized. This leads to the accumulation of 2-DG-6-P, resulting in product inhibition of hexokinase and subsequent inhibition of glycolysis [17].

## Materials and methods

### Yeast strains, plasmids, and growth conditions

*S. cerevisiae* yeast strains were grown in YPD (2% peptone, 1% yeast extract, 2% glucose) or yeast nitrogen base minus uracil (AA-Ura) media, to prevent plasmids loss at 30 °C [19]. Yeast strains used in this study are listed in Supplemental Table 1. Plasmids were transformed into yeast strains using the standard lithium acetate transformation method [20]. AA-Ura or YPD plates containing 2% glucose were supplemented with 0.2% w/v 2-DG. To prevent plasmid loss and ensure that only vector-containing cells can grow, yeast strain transformants are grown on selective media (AA-URA).

### Mammalian cell culture

Human prostate cancer cell line, PC-3 cells were cultured in F-12 K Medium (Kaighn’s Modification of Ham’s F-12 Medium) supplemented with 10% Fetal Bovine Serum (FBS) and 1% penicillin-streptomycin. All cells were cultured in 37 °C, 5% CO2 incubator.

### *HXK* cloning and yeast strain construction

To amplify *yHXK1* and *yHXK2* from yeast genomic DNA, we employed specific primer pairs (Kpn1-*HXK1*/Xba1-*HXK1* and Kpn1-*HXK2*/Xba1-*HXK2*; primer sequences provided in Supplemental Table 2). Following amplification, the resulting DNA fragments were gel-purified using a Qiagen gel purification kit and subsequently cleaved with *Kpn1* and *Xba1* restriction enzymes. The resulting fragments were inserted into the yeast expression vector PSF-TEF1-URA3 (OGS534, Milipore-Sigma) at the corresponding restriction enzyme sites.

The yeast-codon optimized human *HK* genes *hHK1* and *hHK2* were synthesized directly, with the sequences described in the Supplemental Data. These gene fragments were enzymatically cut using the restriction enzymes *EcoRV* and *XhoI* and integrated into the PSF-TEF1-URA3 vector, utilizing the matching restriction enzyme sites.

To introduce the *HXK*-containing vectors and vector controls into yeast, we followed the standard lithium acetate transformation protocol. Colonies were selected on yeast synthetic complete supplement mixture minus uracil (SC-Ura) plates.

### ATP measurements

Yeast cells (2 OD units at 600 nm) were collected in a 1.5 ml microcentrifuge tube by centrifugation at maximum speed for 1 min and washed once with cold distilled water. The supernatant was removed, and 0.75 ml of 90% acetone was added to the pellet. The resuspended cells were then heated at 90 °C for 15 min to allow the acetone to evaporate, leaving approximately 50 µl of solution. Assay buffer (10 mM Tris, pH 8.0, and 1 mM EDTA) was added to bring the final volume to 500 µl. ATP concentration was measured using the ATP Determination Kit (A22066, Thermo Scientific Inc.) according to the manufacturer’s instructions, with a luminescence microplate reader (BioTek U.S.).

### Immunoblotting assay

To monitor the levels of histone marks in yeast strains, we performed whole cell extraction using tris-buffered saline (20 mM Tris pH 7.5, 150 mM NaCl) with protease inhibitor (phenylmethylsulfonyl fluoride PMSF). Briefly, log-phase cells (5 OD600) were harvested by spinning cultures at 5000 rpm (revolutions per minute) then washing the pellets with water, heating them at 95° C for 3 min, and suspending them in 50 ul tris-buffered saline buffer with 2 mM PMSF. Cells were physically broken by glass bead–beating them for 2 cycles (30 s per cycle). Proteins were then solubilized in 50 ul 2x SDS sample buffer [Tris-Cl (pH 6.8), 4% (w/v) sodium dodecyl sulfate (SDS; electrophoresis grade), 0.2% (w/v) bromophenol blue, 20% (v/v) glycerol]. Insoluble debris was separated from supernatant and removed by spinning the samples at the 15,000 g speed. To prepare mammalian cell lysates, cells were washed with 1x PBS and cells were resuspended in RIPA buffer (50 mM Tris-Cl (pH 8.0), 150 mM sodium chloride, 1% NP-40, 0.5% deoxycholate, 0.1% SDS). Protease inhibitors were added and kept on ice for 10 min. Soluble proteins were extracted by high-speed centrifugation. Soluble proteins (the supernatant) were separated by SDS-PAGE, transferred to nitrocellulose membrane (Bio-Rad 1620115) using Tris-Glycine buffer and probed with different primary antibodies [anti-γ-H2A antibody (ab15083 Abcam), tubulin (6A204 Santa Cruz), human hexokinase 2 (C64G5 Cellsignaling), H3K4me3 (ab8580 Abcam), Anti FLAG (F1804 Sigma), H3K36me3 (ab9050 Abcam), H3K56Ac (mc1681 provided by Dr. Zhiguo Zhang [21]), Sir2 (provided by Dr. Zhiguo Zhang) [22], H3K27Ac (07-360 Millipore), and H3K5,8,12Ac (C15410021 Diogenode), Hexokinase 2 (NBP2-44234, Novusbio), PGK1 (22C5D8 Abcam)]. The stain-free gel or Sir2, PGK1 is used as the loading control. The second antibody is either goat anti rabbit IgG HRP (AB_2337913, Jackson Immnoresearch) or goat anti mouse IgG HRP (AB_10015289, Jackson Immnoresearch). Immunoblots were developed by using Chemiluminescent Substrate (PI37069, Thermo-Scientific)and images were acquired using Bio-Rad Chemidoc (12003154, Bio-Rad).

### Cell cycle analysis

Yeast strains were grown in YPD medium at 30° C until the cells reached mid-log phase (∼ 0.6 OD at 600 nm), at which time they were treated with 2-DG (0.2% w/v). A total of ∼ 1 × 10^7^ cells were harvested at various time points by centrifugation, washed with water, and fixed with 95% ethanol overnight at 4° C. Fixed cells were collected by centrifugation, washed with 50 mM sodium citrate buffer (pH 7.5), and resuspended in 500 µL sodium citrate buffer (pH 7.5) with RNAase A (0.025 mg/mL). After cells were incubated for 1 h at 50° C, 25 µL proteinase K (20 mg/mL) was added to the cell suspension and the mixture was incubated at 50° C for another hour. Cells were subsequently stained overnight at 4° C using propidium iodide at a final concentration of 0.02 mg/mL. Finally, samples were analyzed in a fluorescence-activated cell sorting (FACS) flow cytometer (BD Fortessa cytometer). A minimum of 50,000 cells per sample were acquired.

### Analysis of silencing-loss at the *HML (HoMothallism Left)* locus using the CRASH assay

The yeast strains WT (cyc1250), *hxk2Δ* (cyc1253), WT + *PSF-yHXK2* (cyc1247), and *hxk2Δ + PSF-yHXK2* (cyc1251) were used to measure the apparent silencing-loss rate at the *HML* locus. Briefly, 10 colonies of each strain were grown separately overnight in SC media, diluted to 0.01 OD at 600 nm in SC media, and grown for 5 h at 30° C. Cells were treated with 20 µM nicotinamide for the green fluorescent protein (GFP) + control; cells were grown in hygromycin (200 µg/ml) for the red fluorescent protein (RFP) + control. The apparent silencing-loss rate at the *HML* locus was calculated by dividing the number of RFP + GFP + cells (cells that have recently undergone Cre-mediated recombination express GFP but not RFP) by the total number of cells with the potential to lose silencing (RFP + GFP- and RFP + GFP+). For each colony, 50,000 events were analyzed using a BD Fortessa cytometer.

### Enrichment and sequencing of protein-associated nascent (eSPAN)

The yeast strains WT (cyc1023), *hxk2Δ* (cyc1025), WT + PSF-*HXK2* (cyc1022), and *hxk2Δ* + PSF-*HXK2* (cyc1024) were used in this experiment. Briefly, yeast cells were grown in SC-URA- medium to exponential growth phase (∼ 0.5 OD at 600 nm). Two doses of α factor (5 µg/ml; EZBiolab) were added for 3 h at 25° C to arrest cell growth at the G1 phase. Cells were washed twice with cold water and then released into fresh YPD medium containing 400 mg/L Bromodeoxyuridine (BrdU) and 200 mM hydroxyurea for 45 min at 30° C. Hydroxyurea stalls the replication fork but does not interfere with assembly of newly synthesized and parental histones [23–25].

Cells were fixed by adding freshly prepared paraformaldehyde (final concentration 1%) at 25° C for 20 min, followed by quenching with 0.125 M glycine for 5 min at room temperature. After fixation, cells were washed twice with cold water and collected by centrifugation at 3000 rpm for 5 min. For chromatin immunoprecipitation (ChIP), cells were washed and lysed in 0.1 ml ChIP lysis buffer [50 mM HEPES (pH 8.0), 150 mM NaCl, 2 mM EDTA, 1% Triton X-100, 0.1% sodium deoxycholate] with glass beads. Broken cells were collected and washed twice with NP buffer [1.6 M sorbitol, 2 mM CaCl2, 5mM MgCl, 50 mM NaCl, 14 mM b-mercaptoethanol, 10 mM Tris-HCl (pH 7.4), 0.075% NP-40, 5 mM spermidine]. Chromatin was digested primarily to di- and mononucleosomes using the proper amount of MNase (LS004797, Worthington, ∼ 1 unit) at 37° C for 20 min. The digestion was terminated with 5 µl 0.5 M EDTA and 90 µl 5X ChIP lysis buffer and kept on ice for 30 min. Next, cells were lightly sonicated for five cycles (Bioraptor Pico machine, 30 s ON/OFF) at 4 °C to release chromatin fragments into solution. Soluble chromatin was immunoprecipitated with anti-H3K4me3 antibody (ab8580 Abcam) [21]. Prewashed Protein G Sepharose beads (17-0618-02, GE Healthcare) were used to recover the immunoprecipitated chromatin. After washing the beads extensively, ChIP DNA was recovered using the Chelex-100 protocol [15, 26–28].

ChIP DNA was denatured by incubating it at 100° C for 5 min and then on ice for 5 min. DNA was diluted with BrdU IP buffer [1X PBS, 0.0625% Triton X-100 (v/v)]. BrdU antibody (0.17 µg/ml; 555627, BD Biosciences) was added, and samples were incubated at 4° C for 2 h. Next, 20 µl prewashed Protein G beads (17-0618-02, GE Healthcare) were added to each sample and incubated for an additional hour at 4° C. The beads were extensively washed, then DNA was eluted with 100 µl 1X TE buffer containing 1% SDS and purified using a QIAGEN MinElute PCR Purification kit. Single-stranded DNA libraries were prepared using an Accel-NGS 1 S Plus DNA library kit (10096, Swift Biosciences).

### Sequence mapping and data analysis

Sequence mapping, nucleosome mapping, and eSPAN analysis were performed similarly to what has been previously described [23, 24]. Briefly, reads were mapped to the *Saccharomyces* Genome Database (http://www.yeastgenome.org/) reference genome with Bowtie2 software [29]. Only paired-end reads with both ends mapped correctly were selected for continued analysis. We determined nucleosome occupancy using 120–170 bp DNA fragments calculated from paired-reads, using Python programs we developed ourselves. To calculate the eSPAN bias pattern, we separated forward (Watson strand) and reverse (Crick strand) reads following the reference genome. Nucleosome positions around DNA replication origins were determined previously [30]. Total eSPAN sequence reads at ± 10 nucleosomes surrounding the DNA replication origins were counted. The log2 ratio of Watson over Crick strand reads at each nucleosome position was used to obtain the average eSPAN bias pattern.

### Western blot to detect yeast Rad53-P and γH2AX

The sample collection and process with Rad53-P and γH2AX followed previous publication [31]. Blotting was then performed with anti- Rad53 antibody (ab104232, Abcam) and γH2A.X (phospho S139) (ab11174 Abcam).

## Results

### Human *HK1* or *HK2* cannot complement yeast HXK deletion mutant as determined by yeast cell growth and 2-DG toxicity

**Fig. 1:**
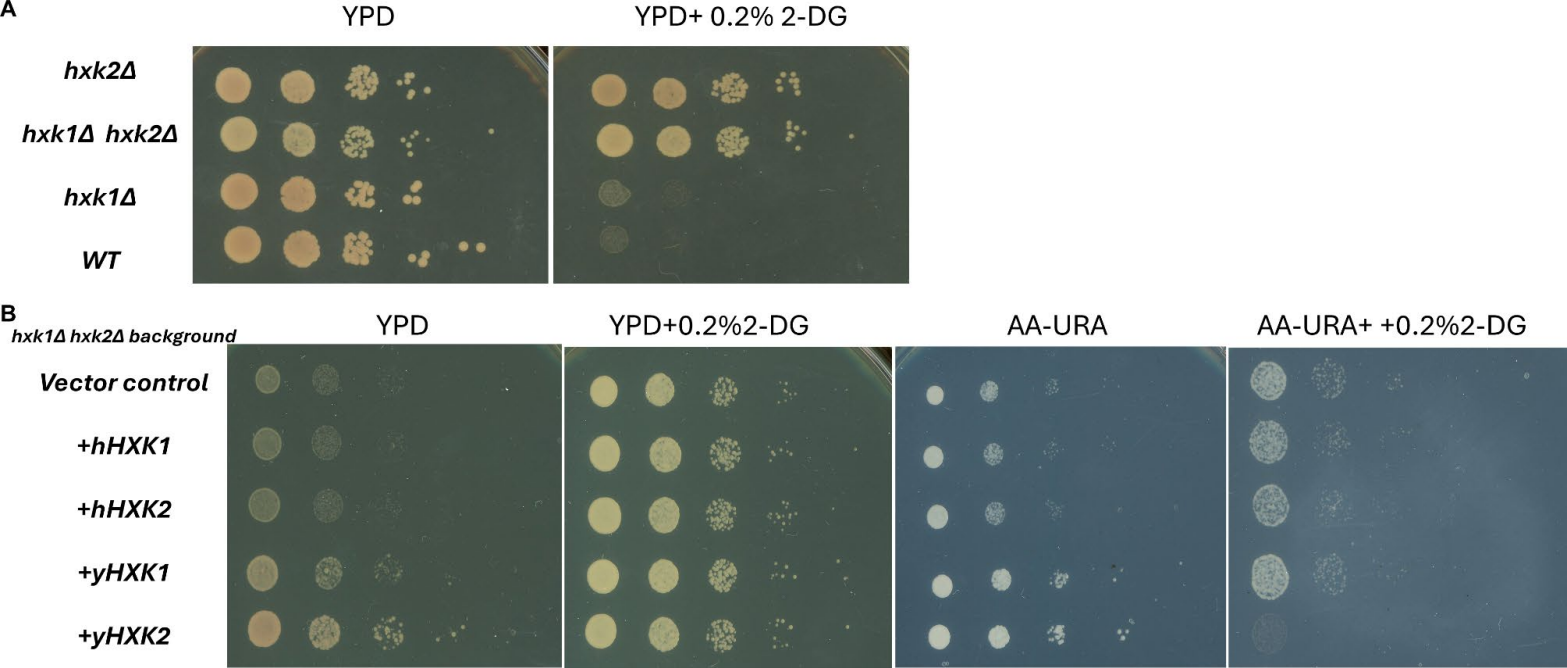
In yeast, *HXK2* is the dominant hexokinase; overexpressing yeast *HXK1* or *HXK2*, but not human *HK1* or *HK2*, complements the slow cell growth phenotype in yeast cells lacking *HXK1* or *HXK2*. (**A**) Ten-fold serial dilutions of yeast cells of the indicated genotypes were plated onto YPD medium with or without 0.2% 2-deoxy-D-glucose (2-DG, an HXK2 inhibitor). This result suggests that *HXK2* is the dominant player in glucose metabolism. (**B**) Yeast *HXK1* or *HXK2* can complement the cell growth phenotype in *hxk1Δ hxk2Δ* cells, but human *HK1* and *HK2* cannot. Ten-fold serial dilutions of yeast cells of the indicated genotypes were plated onto YPD medium with or without 0.2% 2-DG (nonselective for the plasmid containing the *HXK* genes) and AA-URA medium with or without 0.2% 2-DG (selective for the plasmid). These results indicate that 2-DG is more specifically toxic towards targeting *HXK2* than *HXK1* because Hxk2 has a higher affinity to 2-DG than glucose (Glc), but Hxk1 has a higher affinity to Glc [32]

In yeast, two *HXK*s, *HXK1* and *HXK2*, and one glucokinase (*GLK1*) phosphorylate glucose, the first irreversible step in the intracellular metabolism of glucose [11]. Among these three enzymes, *HXK2* is the dominant player in cell growth, as evidenced by the slow growth phenotype exhibited by *hxk2Δ* and *hxk1Δ*, *hxk2Δ* mutant cells; the growth of *hxk1Δ* cells is comparable to that of wild-type (WT) cells (Fig. 1A, *left*). Importantly, as shown previously [32], the glucose metabolic inhibitor 2-DG readily targets *Hxk2* but not *Hxk1* (Fig. 1A, *right*). This conclusion was based on the observation that the 2-DG show more toxic effect on *hxk1Δ* cells, than in *hxk2Δ* cells (Fig. 1A, *right*).

To investigate the effects of *HXK* expression, we overexpressed both yeast *HXK*s (y*HXK1*, y*HXK2*) and their human orthologs (h*HK1*, h*HK2*) in *hxk1Δ hxk2Δ* double mutant yeast cells. The *HXK* genes were placed under the control of the yeast constitutive *TEF1* promoter [21] in a plasmid, and we grew each transformed strain under conditions that selected for the plasmid (AA-URA medium) and did not select for the plasmid (YPD medium). We hypothesized that expressing both human and yeast versions of the *HXK* genes could complement the slow growth phenotype of *hxk1Δ hxk2Δ* double mutant. Surprisingly, overexpressing human *HKs* in the yeast mutants cultured in YPD or AA-URA media had no discernible impact on yeast proliferation relative to vector-only control yeast (Fig. 1B). This outcome strongly suggests that human *HK1* and *HK2* do not function effectively in yeast cells. By contrast, expression of yeast *HXK1* and *HXK2* exhibited a complementary effect on yeast proliferation, evident in both YPD and AA-URA media (Fig. 1B). A plausible explanation for the lack of function of human hexokinases in yeast is the feedback inhibition by Glucose-6-Phosphate (Glc-6-P). Unlike yeast hexokinases, which are inhibited by Trehalose-6-Phosphate (T6P), human hexokinases are sensitive to inhibition by Glc-6-P [33]. At here, we want to explain a little about the media difference. Because cells could frequently lose the vectors (HXKs-containing or empty control) during proliferation process, the cells on the non-selective media (YPD) are mixture of cells with or without vectors. On the selective media (AA-URA), only vectors containing cells can grow.

Next, we investigated the toxic effect of 2-DG on overexpressed *HXK*s. The purpose is to further test the 2-DG’s specificity on the *Hxk*s. When the transformed yeast strains were cultured on YPD supplemented with 0.2% 2-DG (non-selective media with a glucose metabolic inhibitor), no significant differences in proliferation were observed relative to the vector control strain. The loss of the transformed plasmid in the non-selective conditions is the cause of this finding. The strains were cultivated on AA-URA + medium supplemented with 0.2% 2-DG (selective media with an inhibitor of glucose metabolism). Because Hxk2 is more active than Hxk1, the inhibitory effect of 2-DG was more specific to cells containing yHXK2 than yHXK1(Fig. 1B). This finding emphasizes the critical function of yeast HXK2 in glucose metabolism and the inhibitory action of 2-DG on yHxk2p.

### Impact of *HXK2* expression on global histone modification levels

**Fig. 2:**
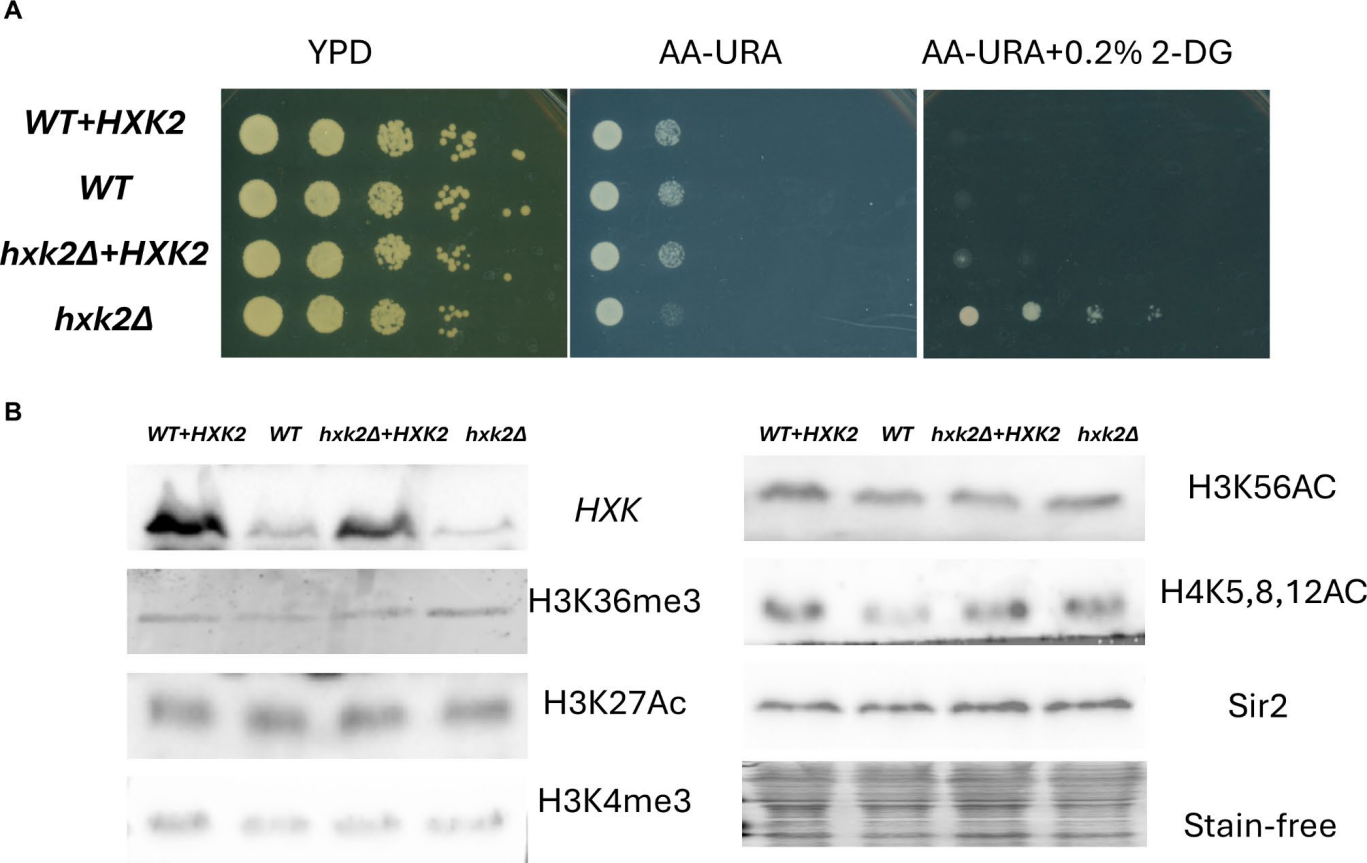
Impact of *HXK2* expression on yeast cell growth and total histone modification levels. In this panel, we confirm the *HXK*s expression level by western blot and test *HXK*s level on total histone modification levels. (**A**) Cell growth rate of WT, WT + *HXK2*, *hxk2Δ* + *HXK2*, and *hxk2Δ* yeast strains. Ten-fold serial dilutions of yeast cells were plated onto YPD medium and AA-URA (conditions selective for the vector containing cells) with or without 0.2% 2-deoxy-D-glucose (2-DG, an *HKs* inhibitor) medium. The purpose of these dot assay is phenotype confirming of the genotypes. (**B**)Histone modification (H3K36me3, H3K27Ac, H3K4me3, H3K56Ac and H4K5,8,12Ac) levels by Western blot analysis. The expression levels of the four yeast strains were determined using a yeast HXKs-specific antibody. This antibody recognizes both Hxk2 and Hxk1, as illustrated in Fig. 3C. The exposure growth cells on AA-URA medium were collected for Western blot analysis

Overexpression of HXKs can result in the production of additional metabolic byproducts, such as acetyl-coenzyme A (Acetyl-CoA). Acetyl-CoA acts as a substrate for histone acetylation modifications, potentially resulting in changes to chromatin structure [34, 35]. To explore the relationship between *HXK2* expression and the chromatin structure/epigenome, we employed four yeast strains characterized by varying *HXK2* expression levels (ranked highest to lowest): WT + *HXK2*, *hxk2Δ* + *HXK2*, WT, and *hxk2Δ* (Fig. 2). First, we confirmed that expression levels for the strains were as expected (Fig. 2A). While overexpression of yeast HXK2 in the WT strain appeared to have no beneficial effect on cell proliferation under the used growth conditions, it significantly increased proliferation in the *hxk2Δ* yeast strain (Fig. 2A on AA-URA plate), indicating complementation *hxk2Δ*. The results are consistent with our former observation that the yeast *HXK2* expression can complement the yeast *hxk1Δ hxk2Δ* (Fig. 1).

Next, we conducted immunoblot analysis to assess the effects of *HXK2* expression on global histone modifications, as well as the abundance of the major chromatin silencing protein, Sir2, in the four yeast strains [13]. We examined modifications such as H3K36me3 (associated with active transcribed genes), H3K27Ac (associated with active gene transcription), H3K56AC(linked with DNA replication), and H4K5AC (linked to active gene transcription) [36]. Given that HXK2 serves as the rate-limiting enzyme in glucose metabolism and that acetyl-CoA is one of its primary substrates, it’s noteworthy that acetyl-CoA is also a crucial substrate for histone acetylation modifications. Considering this, *HXK2* expression level may affect the epigenome, which may show different histone modification levels. However, no substantial differences were detected in the levels of these histone marks or in the abundance of Sir2 across the four yeast strains (Fig. 2B). This finding indicates *HXK2* expression does not exert global effects on histone tail modifications. It is important to note, however, that these results do not rule out the possibility of specific changes in histone modifications at a particular region, warranting further investigation into the potential context-dependent impact of *HXK2* on histone modifications.

### Impact of *HXK2* expression on parental histone transfer

**Fig. 3:**
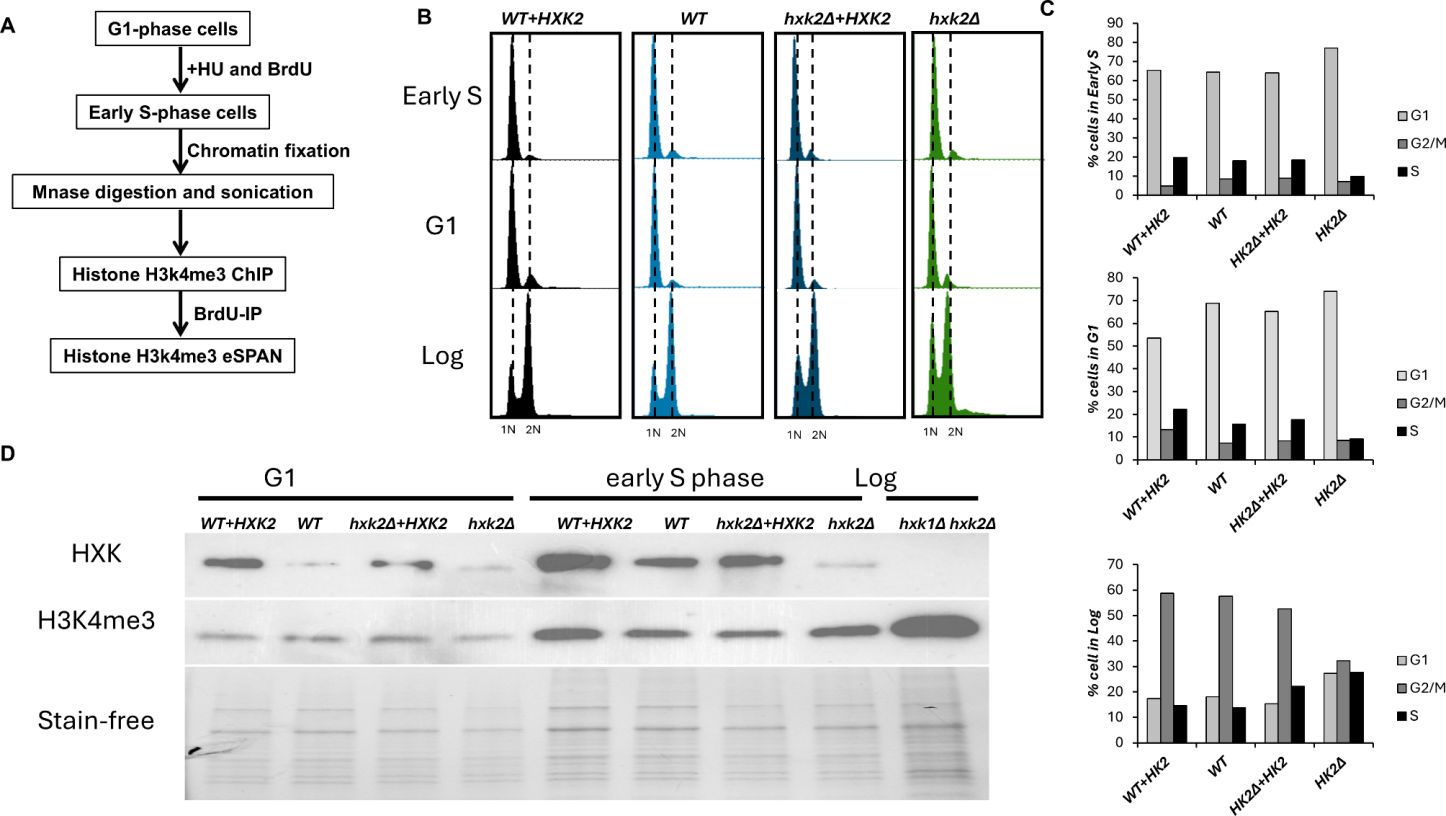
Parental histones transfer experimental procedure: H3K4me3 eSPAN (enrichment and sequencing of protein-associated nascent DNA) analysis. (**A**) Procedure for monitoring the deposition of parental (H3K4Me3) histones at early DNA replication origins in yeast. (**B**-**C**) Fluorescence-activated cell sorting (FACS) analysis monitoring cell cycle progression in WT, WT + *HXK2*, *hxk2Δ* + *HXK2*, and *hxk2Δ* yeast strains. The FACS data indicate that the cell synchronization procedure for eSPAN analysis works well. (**C**) Histograms showing the distribution in the different phases of the cell cycle. (**D**) Western blot analysis monitoring *HXK* and H3K4me3 levels in the yeast strains from (**B**). The log phase *hxk1Δ hxk2Δ* strain sample serves as a negative control for the *Hxk* antibody

One crucial aspect of stable epigenetic inheritance is the successful replication of epigenetic information during DNA replication. Nucleosome assembly, including the appropriate transfer of both parental (recycled histones from mother DNA strand) and newly synthesized histones (de nova synthesized histone), plays a pivotal role in establishment of both euchromatin (transcriptionally active regions) and heterochromatin (transcriptionally silent regions). Given the fundamental step in epigenetic regulation, we investigated whether *HXK2* expression influences parental histone transfer and assembly. In WT yeast, parental H3-H4 tetramers are normally distributed nearly evenly between the leading and lagging strands of DNA replication forks, with a slight bias toward the lagging strand [23]. To assess the impact of *HXK2* expression on parental histone distribution, we conducted an eSPAN assay, employing H3K4me3 as a marker for parental H3-H4 histones, as in our previous studies [15, 37]. Briefly, yeast cells were synchronized at the G1 phase and released into early S phase in the presence of BrdU and hydroxyurea. Cell harvesting occurred 45 min after release (Fig. 3A). Throughout the cell culture process, we employed selective culture medium (AA-URA) to prevent the loss of the *HXK2*-containing plasmid. FACS analysis of DNA content confirmed the effectiveness of our cell synchronization procedure (Fig. 3B, C), and Western blot analysis confirmed the well-controlled expression of *HXK2* (Fig. 3D). In the hypothesis, we would expect to observe the changes of parental histone distribution pattern on the two replicating strands by eSPAN analysis.

**Fig. 4:**
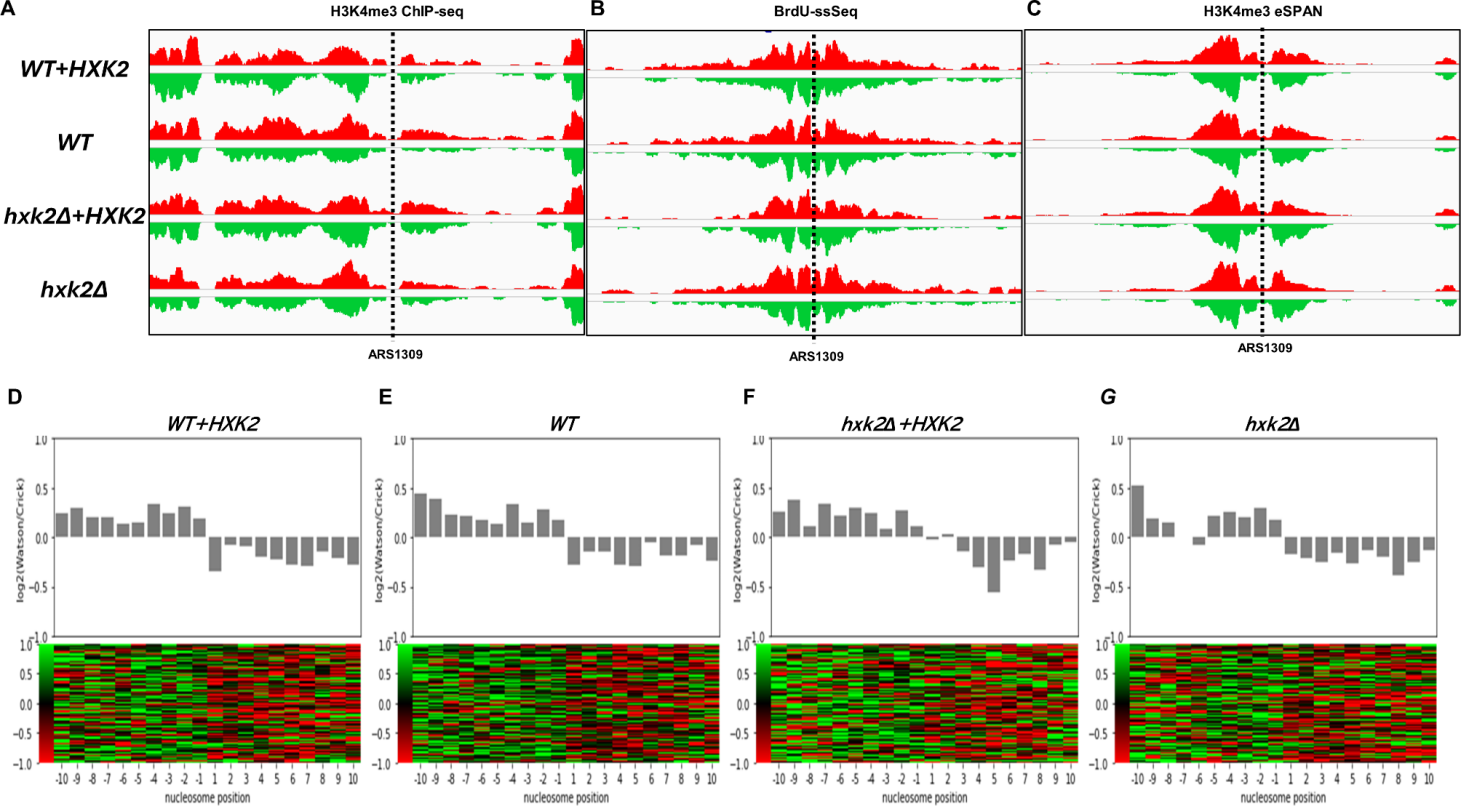
Parental histone H3 (H3K4Me3) eSPAN (enrichment and sequencing of protein-associated nascent DNA) analysis at different *HXK2* expression levels. A snapshot of parental histone (**A**) H3 (H3K4me3) chromatin immunoprecipitation (ChIP), (**B**) BrdU-IP-ssSeq, and (**C**) H3K4me3 eSPAN read enrichment at the early replication origin ARS1309 for the WT, WT + *HXK2*, *hxk2Δ* + *HXK2*, and *hxk2Δ* yeast strains. Sequence reads were mapped to both the Watson (*red*) and Crick (*green*) strands of the reference genome. We did not observe any obvious difference in the BrdU IP-ssSeq, H3K4me3 ChIP-seq and eSPAN. (**D**-**G**) Average bias ratio of parental histone H3 (H3K4Me3) for the WT, WT + *HXK2*, *hxk2Δ* + *HXK2*, and *hxk2Δ* yeast strains. *Top*: Average bias ratio of H3K4Me3 eSPAN peaks at each of the 10 nucleosomes surrounding the 134 early replication origins in the yeast genome. *Bottom*: Heatmaps representing the bias ratio of the same H3K4Me3 eSPAN peaks. Individual nucleosome positions (− 10 to + 10) are indicated relative to the origin. Each row represents the average log2 Watson/Crick ratio of H3K4Me3 eSPAN sequence reads at one origin

H3K4me3 ChIP, BrdU-IP-ssSeq, and H3K4me3 eSPAN sequence data were mapped to both the Watson and Crick strands of the yeast reference genome. No substantial differences were observed among the four strains for H3K4me3 ChIP and BrdU-IP-ssSeq data at typical mapping sites, such as ARS1309 (Fig. 4A and B). H3K4me3 eSPAN peaks at ARS1309 (Fig. 4C) displayed a nearly symmetric bias pattern, with a slight preference for the lagging strand in all four yeast strains. These findings suggest that parental histones H3-H4 were distributed in a nearly symmetrical manner between the leading and lagging strands at hydroxyurea-stalled replication forks, regardless of *HXK2* expression levels.

To determine whether the results we obtained at ARS1309 were characteristic of replication origins around the genome, we calculated the average strand bias ratio of H3K4me3 eSPAN peaks surrounding all 134 early DNA replication origins in yeast. The average bias ratio reflects the relative abundance of histones on the leading vs. lagging strand. Consistent with our findings for H3K4me3 eSPAN data at ARS1309 (Fig. 4C), all four strains exhibited a bias toward the lagging strand at these early DNA replication origins, regardless of *HXK2* expression levels (Fig. 4D-G). However, we did observe significant variation in the average strand bias ratio at different nucleosome locations around the replication origins, particularly in the *hxk2Δ* strain (Fig. 4F, G). This variability may be attributable to differences in cell growth rates or low BrdU incorporation rates, factors that can significantly influence eSPAN bias calculations [37]. Overall, we conclude *HXK2* expression has only a minor effect on the parental histone transfer process.

### Effect of *HXK2* expression and inhibition on chromatin stability

Even the *HXK2* expression level does not have any clear impact on the parental histone transfer process by, it could affect the chromatin stability by other ways. To test this possibility genetically, we performed a well-established CRASH (Cre-reported altered states of heterochromatin) assay to monitor the transient loss of heterochromatin silencing at the *HML* locus. In this assay, the Cre recombinase gene is inserted into the transcriptionally silent *HML* locus. This assay allows for the detection of temporary loss of gene silencing at the *HML* locus by observing the expression of the Cre gene, which causes site-specific recombination at LoxP sites. This results in yeast strains exhibiting GFP sectors. Loss of gene silencing at *HML* locus leads to an increase in switch from RFP to GFP.

**Fig. 5:**
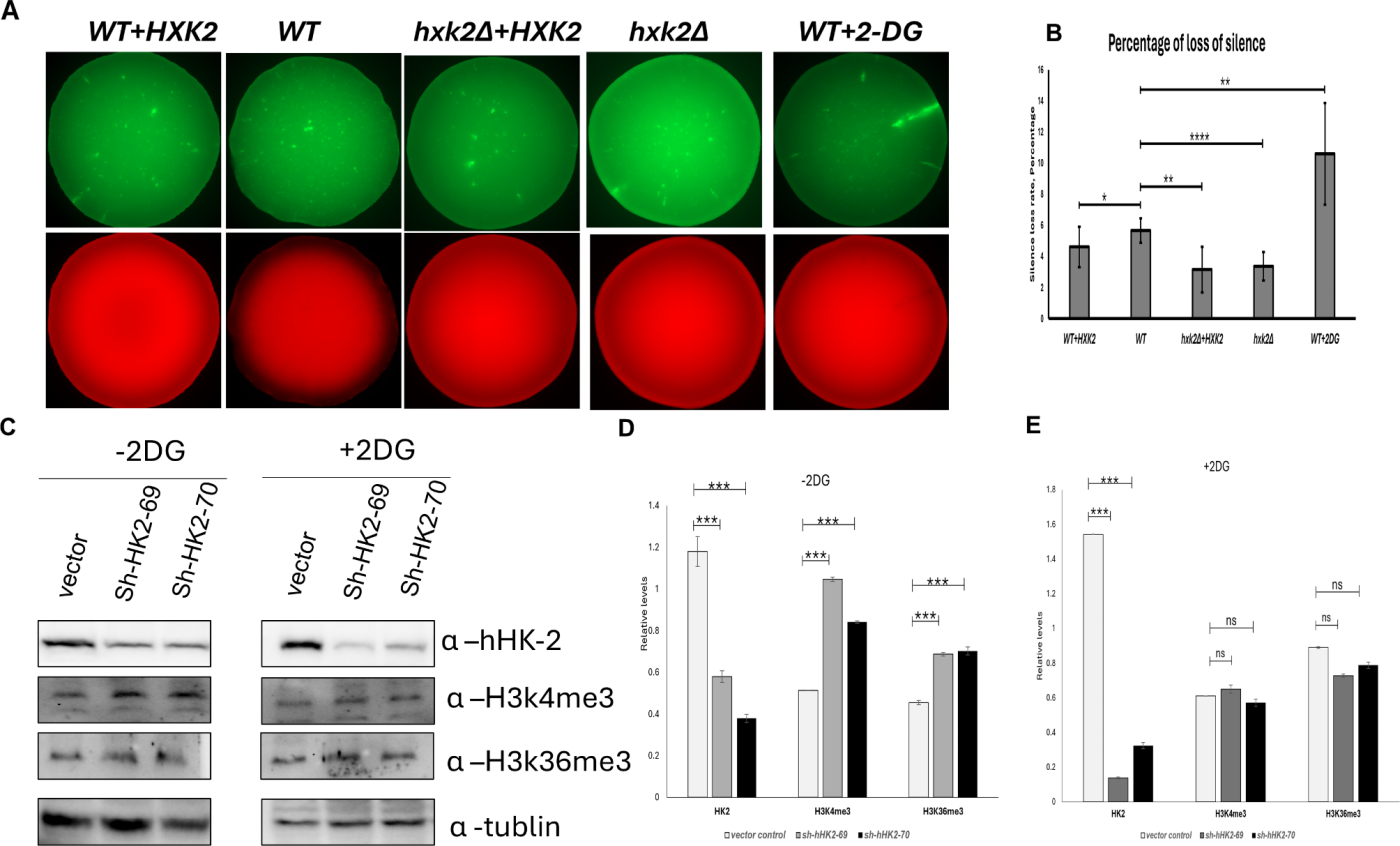
Effect of Hxk levels on loss of silencing at the *HML* locus. (**A**) Typical fluorescence images of colonies derived from WT, WT + *HXK2*, *hxk2Δ* + *HXK2*, *hxk2Δ* yeast strains containing the LoxP-RFP-LoxP-GFP cassette at URA3 locus and a HMLα::cre at the silenced HML locus. The CRASH (Cre-reported altered states of heterochromatin) system [41] was used in this assay. Bright green sectors in GFP channels or dark sectors in RFP channels represent loss of silencing. (**B**) The WT, WT + *HXK2*, *hxk2Δ* + *HXK2*, and *hxk2Δ* yeast strains showed different degrees of silencing at the *HML* locus when analyzed by the CRASH (Cre-reported altered states of heterochromatin) assay. Error bar = standard error. *p < 0.05, **p < 0.01, and ****p < 0.0001 by Student’s t-test. (**C**) Immunoblot analysis of histone marks H3K4me3 and H3K36me3 from lysates prepared from untreated (left panel) and 2-DG treated (right panel) PC-3 cells transduced with two shRNAs targeting HK2. (**D** and **E**) Quantified relative levels of histone marks from panel (**C**) images using ImageJ.

Flow cytometry analysis was utilized to quantify the rate of silencing loss in each of the four yeast strains (Figs. 2, 3 and 4). We hypothesized that overexpression or deletion of *HXK2* changes chromatin stability because of the glucose metabolism alteration. *HXK2* overexpression (WT + *HXK2*) and deletion (*hxk2Δ* or *hxk2Δ* + *HXK2*) both resulted in a lower silencing loss rate than observed in the WT strain (Fig. 5A, B). When WT strains were treated with 2-DG to inhibit glycolysis process, the silencing loss rate increased significantly by student t-test (Fig. 5B). In the experiment, we also assessed whether the other Hexokinases deletion mutants (*hxk1Δ* and *glk1Δ*) affect chromatin stability. The results show no significant difference between *hxk1Δ*,* glk1Δ*,* hxk1Δ glk1Δ* and WT (Supplemental Fig. 1). In the next step, we perform immunoblot analysis to measure the total level of histone post translation modifications in order to understand the effect of 2-DG on chromatin structure using both yeast and human prostate cancer cells (PC-3). In line with the earlier research, we found that all histone modifications were significantly reduced in HXK2 expression strains with 2-DG treatment [38, 39] comparing to *hxk2* deleted yeast cells (Supplemental Fig. 2). We transduced the *sh*-RNAs to deplete the HK2 levels in prostate cancer cells PC-3 cells as we did before [40]. Knockdown of HK2 levels leads to a significant increase in H3K4me3 and H3K36me3 histone levels (Fig. 5C and D). However, no significant difference was observed between normal and HK2 knockdown cells on both levels of H3K4me3 and H3K36me3 histones under 2-DG treatment condition (Fig. 5C and E). These findings suggest a link between the glycolysis process mediated by *HXK2* and chromatin structure. In summary, our results indicate that altered *HXK2* levels reduce yeast chromatin instability and 2-DG treatment can increase chromatin instability. They also shed light on the potential of 2-DG to modulate chromatin dynamics in yeast cells, underscoring the multifaceted effects of this glucose metabolism inhibitor on cellular processes.

### Impact of *HXK2* inhibition on cell cycle progression and the DNA damage response

**Fig. 6:**
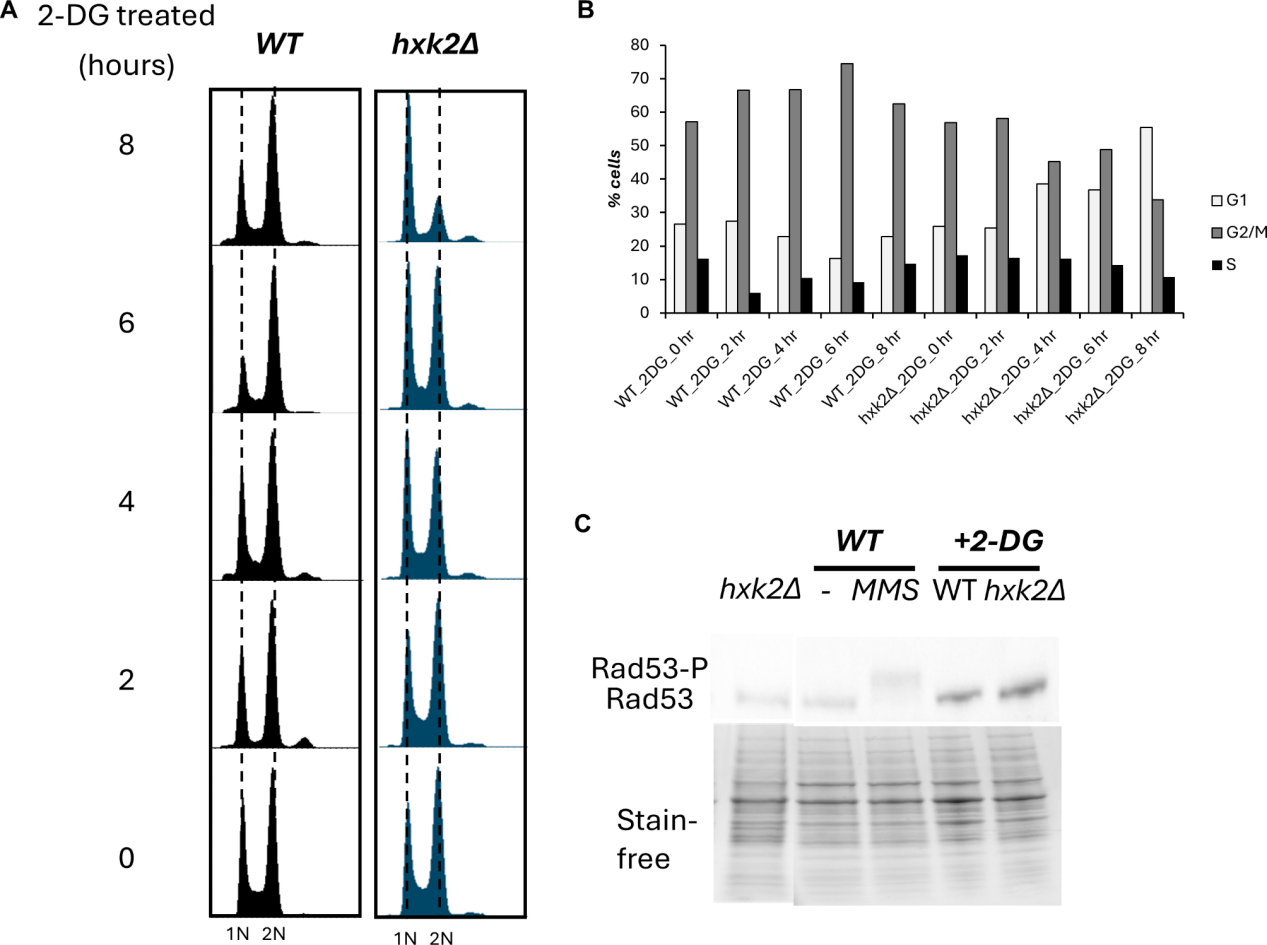
Hxk inhibition arrests yeast cells at G2 phase but does not activate the cell cycle checkpoint. (**A**-**B**) Fluorescence-activated cell sorting (FACS) analysis of DNA content in WT and *hxk2Δ* yeast strains treated with 0.2% 2-deoxy-D-glucose (2-DG, an Hxk2 inhibitor) at different time points. This data indicates that 2-DG treatment leads to more G2 cells. (**B**) Histograms showing the distribution in the different phases of the cell cycle. (**C**) Immunoblot analysis of levels of Rad53-P, a cell cycle checkpoint protein and marker of the DNA damage response, in WT and *hxk2Δ* yeast strains. WT yeast cells before and after treatment with methyl methanesulfonate (MMS; a DNA damaging agent) serve as negative and positive controls, respectively. For 2-DG-treated samples, WT and *hxk2Δ* cells were treated with 0.2% 2-DG for 2 h

From the FACS analysis (Fig. 3B and C), HXK2 deletion appears to increase the S phase population. To investigate potential changes in cell cycle cyclin levels, we generated yeast strains with FLAG-tagged CLN2 (G1-S cyclin) and CLB1(G2-M cyclin). The two cyclin levels were measured using Western blot analysis. The results showed that yeast cells lacking *HXK2* and wild-type yeast cells exhibited similar levels of CLN2 and CLB1 cyclins (Supplemental Fig. 3). This result may be due to the low sensitivity of the method, which might not be sufficient to detect small differences. To gain further insights into the effects of 2-DG on cell physiology, we assessed its influence on cell cycle progression and its potential to induce systemic DNA damage. First, we investigated the effects of *HXK2* inhibition on cell cycle progression. Logarithmically growing yeast cells (both WT and *hxk2Δ*) were subjected to 2-DG treatment, and we conducted FACS at various time points to measure changes in DNA content. We hypothesized that the cell cycle is predominately arrested at one of cell cycle stages because the glucose metabolism is inhibited. In the WT yeast strain, characterized by normal *HXK2* expression, 2-DG treatment predominantly led to cell cycle arrest at the G2 phase (Fig. 6A, B). No significant effects were observed in *hxk2Δ* yeast cells following 2-DG treatment; the G1 enrichment observed in this strain at the 8-hour time point was attributed to prolonged culture and nutrient depletion.

Furthermore, the 2-DG treatment changed the cell cycle progress, which is frequently associated with DNA damage response. We next investigated whether inhibiting *HXK2* induced systemic DNA damage. Both γH2A.X and Rad53(vital cell cycle checkpoint protein) are activated and phosphorylated (H2A (p-S139, p-Rad53) in response to DNA replication stress [42]. Thus, phosphorylated H2A and RAD53 indicates a systemic DNA damage response [43]. For instance, exposure to methyl-methanesulfonate induces detectable levels of p-Rad53 (Fig. 6C). We analyzed p-Rad53 levels in strains treated with 2-DG via Western blot analysis. Our results indicate that neither 2-DG-treated WT nor 2-DG-treated *hxk2Δ* yeast strains displayed activated Rad53 (Fig. 6C). And we analyzed the γH2A.X levels, did not find change in the levels wild type and *hxk2Δ* yeast strains (Supplemental Fig. 3). These findings suggest that 2-DG-induced cell cycle arrest occurs primarily at the G2 phase but does not lead to widespread DNA damage. These observations provide valuable insights into the specific impact of 2-DG on cell cycle dynamics and establish the absence of a prominent DNA damage response, further contributing to our understanding of the cellular effects of this glycolytic inhibitor.

## Discussion

In this study, we explored the impact of altering expression of the glycolytic enzyme *HXK2* on parental nucleosome transfer and epigenetic stability in yeast. The nucleosome assembly primary pathway involves in epigenetics inheritance mechanisms. Epigenetic enzymes employ several metabolic intermediates as substrates or co-factors to carry out post-translational modifications of DNA and histones [44], and numerous studies have underscored the crucial role of histone tail acetylation in nucleosome assembly [45, 46]. Disrupting acetylation modifications can result in genome instability [45, 46]. Thus, we hypothesized that altering *HXK2* expression might impact glycolysis rates in the cell [47, 48] and thus the cell’s global epigenetic signature and genome stability. However, our findings indicate that changes in *HXK* expression, whether resulting from overexpression or deletion, have a minimal effect on parental nucleosome assembly and seem to decrease chromatin instability. Surprisingly, inhibiting *HXK2* via treatment with 2-DG increased chromatin instability.

These findings imply that in yeast cells with *HXK2* deletions, the minimal ATP levels generated through glycolysis, driven by alternative *HXK*s, such as *HXK1* or *GLK1*, are sufficient to sustain epigenetic stability. Interestingly, recent research has shown that a high-glucose culture medium can enhance chromatin instability, with the NAD-dependent histone deacetylase Sir2 playing a role in this process [49]. It appears that energy source (glucose) availability, rather than enzyme levels, is the primary rate-limiting factor for chromatin stability.

The proliferation of many cancer cells heavily relies on aberrant energy metabolism, such as the Warburg effect or aerobic glycolysis [50]. Therefore, inhibiting Warburg effect has emerged as a potential strategy to target and eliminate tumor cells, with the glycolysis inhibitor 2-DG being one potential candidate drug of this type. As mentioned, our study suggests that 2-DG treatment increases epigenetic instability. However, this effect appears to occur independent of glycolysis inhibition, as lower *HXK2* levels increase epigenetic stability. It’s known that 2-DG can drains the cells ATP through accumulation of non-metabolizable 2-DG-6P [51]. As a result of ATP depletion, various cellular processes may be affected, potentially leading to chromatin instability. We measured cellular ATP levels in our strains. Overexpression of HXK2 led to an increase in ATP levels, while chronic 2-DG treatment significantly decreased ATP levels. However, HXK2 deletion did not significantly alter cellular ATP levels. Prolonged, moderate 2-DG treatment resulted in only a slight decrease in ATP levels (Supplemental Fig. 4) [52]. Thus, the precise mechanism underlying 2-DG’s impact on chromatin stability warrants further investigation.

Previous report shows that the human GlkB (h*HK*4) protein, which has a similar molecular weight as the yeast *HXK2* protein, can complement the growth phenotype of a yeast *hxk1Δ*/*hxk2Δ*/*glk1Δ* mutant [53]. Likewise, several plant *HXK*s have been shown to complement growth phenotypes in yeast *HXK* mutants [27, 54, 55]. In our experiments, despite effectively functioning yeast *HXK1* and *HXK2* genes within the same vector, overexpressing the human-derived *hHK1* and *hHK2* genes did not rescue the growth defect phenotype of a yeast *hxk1Δ hxk2Δ* mutant. A likely explanation for this outcome is that human HKs are inhibited by their product Glu6P through feedback inhibition. The Glu6P level in yeast cells significantly exceeds the inhibitor constant K_i_ of hHK1 and hHK2 (0.5–2 mM compared to 0.02 mM, respectively) [2, 56]. Strong evidence supports this explanation is the recent isolated *hHK1* and *hHK2* mutants, which can bypass these inhibition [57]. However, alternative explanations cannot be entirely ruled out.

## Conclusions

Hexokinases (*HXK*s) are the key enzymes regulating glycolysis in cells. Overexpression of *HXK*s has been linked to numerous types of cancers and targeting *HXK*s has been suggested as a potential strategy of cancer therapy. In this study, our result suggests either higher or lower *HXK*s expression level decreases the chromatin instability. However, 2-Deoxy-D-glucose (2-DG), a *HXK* inhibitor, treatment cells show increased chromatin instability with unknown mechanisms.

### Electronic supplementary material

Below is the link to the electronic supplementary material.

Supplementary Material 1

## Data Availability

The datasets generated and analyzed during the current study are available in the GEO repository, GSE 245005.
