# Supplementary material for: The role of hexokinases in epigenetic regulation: altered hexokinase expression and chromatin stability in yeast
Source: Epigenetics Chromatin. 2024 Aug 27;17:27. doi: 10.1186/s13072-024-00551-9 (PMC11348520; doi:10.1186/s13072-024-00551-9)
Supplement: Supplementary file 1 — Supplementary Material 1 [file 13072_2024_551_MOESM1_ESM.docx]

**Yeast strains used in this study**

| **Strain** | **Genotype** |
| --- | --- |
| cyc821 | MATB ade2-1 his3-11 trp1-1 leu2-3,112 can1-100 hk1Δ::KanMX hk2Δ::KanMX pdr1Δ::KanMX+pl(URA3::Tef1) |
| cyc822 | MATB ade2-1 his3-11 trp1-1 leu2-3,112 can1-100 hk1Δ::KanMX hk2Δ::KanMX pdr1Δ::KanMX+pl(URA3::Tef1-hHK1) |
| cyc824 | MATB ade2-1 his3-11 trp1-1 leu2-3,112 can1-100 hk1Δ::KanMX hk2Δ::KanMX pdr1Δ::KanMX+pl(URA3::Tef1-hHK2) |
| cyc826 | MATB ade2-1 his3-11 trp1-1 leu2-3,112 can1-100 hk1Δ::KanMX hk2Δ::KanMX pdr1Δ::KanMX+pl(URA3::Tef1-yHK1) |
| cyc828 | MATB ade2-1 his3-11 trp1-1 leu2-3,112 can1-100 hk1Δ::KanMX hk2Δ::KanMX pdr1Δ::KanMX+pl(URA3::Tef1-yHK2) |
| cyc739 | MATa ade2-1 ura3-1 his3-11 trp1-1 leu2-3,112 can1-100 hk1Δ::KanMX |
| cyc743 | MATα ade2-1 ura3-1 his3-11 trp1-1 leu2-3,112 can1-100 hk2Δ::KanMX |
| cyc751 | MATB ade2-1 ura3-1 his3-11 trp1-1 leu2-3,112 can1-100 hk1Δ::KanMX hk2Δ::KanMX |
| cyc1022 | MATA ade2-1 ura3-1 his3-11,15 trp1-1 leu2-3,112 can1-100+ LEU2::BrdU–Inc+PSF-TEFI- yHK2-URA3 |
| cyc1023 | MATA ade2-1 ura3-1 his3-11,15 trp1-1 leu2-3,112 can1-100+ LEU2::BrdU–Inc+PSF-TEFI-URA3 |
| cyc1024 | MATA ade2-1 ura3-1 his3-11,15 trp1-1 leu2-3,112 can1-100 hk2Δ::KanMX+ LEU2::BrdU–Inc +PSF-TEFI- yHK2-URA3 |
| cyc1025 | MATA ade2-1 ura3-1 his3-11,15 trp1-1 leu2-3,112 can1-100 hk2Δ::KanMX+ LEU2::BrdU–Inc+PSF-TEFI-URA3 |
| cyc1247 | MatB lys2 his3-11,15 leu2-3,112 can1-100 hml2alpha::cre ura3Δ::GPDpro-loxP-yEmRFP-CYC1term-hphMX-loxP-yEGFP-ADH1term+ + PSF-TEFI-yHK2-URA3 |
| cyc1250 | MatB lys2 his3-11,15 leu2-3,112 can1-100 hml2alpha::cre ura3Δ::GPDpro-loxP-yEmRFP-CYC1term-hphMX-loxP-yEGFP-ADH1term+ PSF-TEFI-URA3 |
| cyc1251 | MatB lys2 his3-11,15 leu2-3,112 can1-100 hml2alpha::cre ura3Δ::GPDpro-loxP-yEmRFP-CYC1term-hphMX-loxP-yEGFP-ADH1term hk2Δ::KanMX+ PSF-TEFI-yHK2-URA3 |
| cyc1253 | MatB lys2 his3-11,15 leu2-3,112 can1-100 hml2alpha::cre ura3Δ::GPDpro-loxP-yEmRFP-CYC1term-hphMX-loxP-yEGFP-ADH1term hk2Δ::KanMX+ PSF-TEFI-URA3 |
| cyc669 | MatA lys2 his3-11,15 leu2-3,112 can1-100 hml2alpha::cre ura3Δ::GPDpro-loxP-yEmRFP-CYC1term-hphMX-loxP-yEGFP-ADH1term |
| sk460 | MatA lys2 his3-11,15 leu2-3,112 can1-100 hml2alpha::cre ura3Δ::GPDpro-loxP-yEmRFP-CYC1term-hphMX-loxP-yEGFP-ADH1term, hk1Δ::KANMX |
| sk461 | MatA lys2 his3-11,15 leu2-3,112 can1-100 hml2alpha::cre ura3Δ::GPDpro-loxP-yEmRFP-CYC1term-hphMX-loxP-yEGFP-ADH1term, glk1Δ::HIS |
| sk462 | Mata lys2 his3-11,15 leu2-3,112 can1-100 hml2alpha::cre ura3Δ::GPDpro-loxP-yEmRFP-CYC1term-hphMX-loxP-yEGFP-ADH1term, glk1Δ::HIS, hk1Δ::KANMX |
| Sk548 | MATA ade2-1 ura3-1 his3-11,15 trp1-1 leu2-3,112 can1-100+ LEU2::BrdU–Inc,CLN2-flag::NATMX, PSF-TEF1-URA |
| Sk549 | MATA ade2-1 ura3-1 his3-11,15 trp1-1 leu2-3,112 can1-100+ LEU2::BrdU–Inc,CLN2-flag::NATMX, PSF-TEF1-yHK2-URA |
| Sk550 | MATA ade2-1 ura3-1 his3-11,15 trp1-1 leu2-3,112 can1-100+ LEU2::BrdU–Inc,CLB1-flag::NATMX, PSF-TEF1-URA |
| Sk551 | MATA ade2-1 ura3-1 his3-11,15 trp1-1 leu2-3,112 can1-100+ LEU2::BrdU–Inc,CLB1-flag::NATMX, PSF-TEF1-yHK2-URA |
| Sk552 | MATA ade2-1 ura3-1 his3-11,15 trp1-1 leu2-3,112 can1-100 hk2Δ::KanMX+ LEU2::BrdU–Inc , CLN2-flag::NATMX, PSF-TEF1-URA |
| Sk553 | MATA ade2-1 ura3-1 his3-11,15 trp1-1 leu2-3,112 can1-100 hk2Δ::KanMX+ LEU2::BrdU–Inc , CLN2-flag::NATMX, PSF-TEF1-yHK2-URA |
| Sk554 | MATA ade2-1 ura3-1 his3-11,15 trp1-1 leu2-3,112 can1-100 hk2Δ::KanMX+ LEU2::BrdU–Inc , CLB1-flag::NATMX, PSF-TEF1-URA |
| Sk555 | MATA ade2-1 ura3-1 his3-11,15 trp1-1 leu2-3,112 can1-100 hk2Δ::KanMX+ LEU2::BrdU–Inc , CLB1-flag::NATMX, PSF-TEF1-yHK2-URA |

**Oligos used in this study**

| **Oligo** | **Sequence** |
| --- | --- |
| Xba1-yHK1 | AAATCTAGATTAAGCGCCAATGATACCAAGAGACTTA |
| Kpn1-yHK1 | AAAGGTACCATGGTTCATTTAGGTCCAAAGAAACCACAGGC |
| Xba1-yHK2 | AAATCTAGATTAAGCACCGATGATACCAACGGACTTACCTTC |
| Kpn1-yHK2 | AAAGGTACCATGGTTCATTTAGGTCCAAAAAAACCACAAGC |
| Kpn1-hHXK2 | AAAGGTACCATGATTGCCTCGCATCTGCTTGCC |
| Xba1-hHXK2 | AAATCTAGACTATCGCTGTCCAGCCTCACGGATGC |
| Xba1-hHXK1 | AAATCTAGATTAGCTGCTTGCCTCTGTGCGTAA |
| Kpn1-hHXK1 | AAAGGTACCATGATCGCCGCGCAGCTCCTGGCC |

**Synthesized yeast-codon optimized human hHK1 fragment**

EcoRV-hHK1HA-XhoI - AAAGATATCATGATTGCAGCCCAGCTTTTGGCATATTATTTTACAGAACTAAAGGATGATCAGGTTAAGAAGATTGATAAGTATCTATACGCGATGCGTTTGAGCGATGAAACACTGATCGACATTATGACACGTTTTAGAAAGGAGATGAAGAATGGGTTGTCTAGGGACTTCAACCCCACGGCTACTGTTAAAATGTTACCGACCTTTGTGAGAAGTATACCCGATGGATCCGAGAAAGGTGACTTCATTGCGCTGGATCTTGGGGGTTCATCCTTTAGAATCCTTCGTGTACAAGTAAACCATGAAAAGAACCAGAATGTCCACATGGAATCAGAAGTGTATGATACCCCAGAAAATATCGTACACGGAAGCGGAAGCCAACTATTCGACCACGTCGCGGAATGTTTAGGGGATTTTATGGAGAAAAGGAAAATCAAAGACAAGAAGTTGCCCGTTGGTTTCACATTTTCTTTCCCTTGCCAGCAGAGTAAAATAGATGAAGCCATTCTGATCACGTGGACCAAGAGGTTCAAAGCGAGCGGAGTGGAAGGGGCGGACGTTGTTAAGTTGTTGAACAAGGCCATCAAAAAGAGAGGCGACTATGACGCAAACATAGTGGCGGTCGTGAATGATACGGTTGGAACAATGATGACGTGTGGGTACGATGATCAACACTGCGAGGTAGGGCTTATCATAGGGACTGGGACAAATGCCTGTTACATGGAGGAACTTAGGCATATCGACTTAGTGGAAGGCGACGAGGGGCGTATGTGTATCAACACTGAATGGGGTGCATTTGGTGACGATGGGAGTTTAGAAGATATTAGAACGGAATTCGATAGAGAAATTGACAGAGGTTCCTTGAATCCAGGCAAACAGCTGTTCGAAAAAATGGTCTCCGGAATGTATCTAGGTGAATTAGTAAGGCTGATTTTAGTCAAAATGGCCAAAGAGGGGTTGCTTTTCGAGGGAAGAATAACCCCCGAGTTGTTGACCAGAGGAAAATTTAATACGAGCGATGTGAGCGCCATAGAGAAAAACAAAGAAGGTTTGCATAATGCTAAGGAAATTCTTACAAGGCTTGGCGTTGAACCGTCAGATGATGATTGTGTCTCAGTGCAGCATGTGTGTACAATAGTGAGTTTTAGATCAGCTAACCTAGTGGCAGCCACTCTAGGAGCAATATTAAACAGACTAAGGGATAACAAAGGTACCCCCAGGCTACGTACTACTGTAGGAGTCGACGGCTCCCTTTATAAAACGCATCCCCAGTACAGCCGTAGATTTCATAAAACTCTGCGTCGTTTGGTTCCAGACTCAGATGTGAGATTTCTACTTTCAGAAAGTGGGAGCGGTAAGGGAGCCGCGATGGTCACTGCCGTGGCATATCGTCTGGCGGAGCAACACCGTCAGATAGAAGAAACGCTGGCACATTTTCATCTTACGAAGGATATGTTGTTAGAAGTTAAGAAAAGGATGAGAGCAGAGATGGAACTAGGCCTGCGTAAACAGACACATAATAACGCCGTAGTAAAGATGCTGCCGTCTTTCGTGAGGCGTACGCCTGATGGCACCGAGAATGGGGACTTTTTGGCTCTTGATTTAGGTGGAACCAATTTCCGTGTCCTGCTAGTCAAAATTCGTTCTGGAAAAAAGAGGACCGTGGAGATGCACAATAAAATTTATGCTATACCTATAGAAATCATGCAGGGCACGGGCGAGGAGTTATTTGACCATATAGTGTCCTGCATTAGCGATTTTTTGGACTACATGGGGATAAAGGGCCCGAGAATGCCACTGGGTTTTACATTTAGCTTTCCTTGCCAGCAGACTAGTTTAGACGCCGGCATTTTGATAACATGGACTAAAGGGTTTAAGGCAACTGATTGTGTGGGGCACGATGTCGTAACTCTATTACGTGACGCCATAAAAAGGCGTGAGGAGTTTGATCTTGATGTTGTAGCGGTTGTGAACGATACAGTGGGTACCATGATGACTTGCGCGTATGAGGAACCCACGTGCGAAGTCGGATTGATTGTCGGTACGGGGAGCAATGCCTGCTATATGGAAGAGATGAAAAACGTGGAGATGGTGGAGGGAGATCAGGGACAAATGTGCATAAATATGGAATGGGGGGCATTCGGTGATAATGGGTGTCTGGATGACATAAGAACACACTATGACCGTCTAGTCGATGAGTACTCACTAAATGCGGGTAAACAAAGGTACGAGAAGATGATTAGTGGCATGTACTTGGGCGAGATTGTTAGAAATATCCTGATCGACTTCACGAAAAAAGGATTTTTATTCAGGGGACAGATCAGTGAGACTCTTAAAACCCGTGGCATTTTCGAAACAAAGTTCCTTTCTCAAATTGAATCAGACCGTCTGGCGCTGCTTCAAGTGAGGGCGATTTTGCAACAGTTGGGATTGAATTCTACGTGTGATGATAGTATCCTTGTCAAGACCGTTTGTGGCGTCGTCTCACGTCGTGCTGCCCAGTTATGCGGTGCTGGCATGGCTGCGGTAGTCGATAAGATTAGAGAAAATAGAGGTTTAGATAGACTTAATGTTACCGTAGGGGTGGACGGTACACTTTACAAACTACATCCACATTTCAGTAGGATAATGCATCAAACAGTCAAAGAACTGAGTCCGAAATGTAATGTATCCTTCCTATTGTCAGAAGATGGATCTGGAAAAGGCGCCGCGTTAATTACTGCCGTAGGGGTGAGATTGAGAACTGAGGCCAGTTCCTACCCATACGATGTTCCTGACTATGCGGGCTATCCGTATGACGTCCCGGACTATGCAGGATCCTATCCATATGACGTTCCAGATTACGCTTGACTCGAGAAA

**Synthesized yeast-codon optimized human hHK2 fragment**

EcoRV-hHK2HA-XhoI - AAAGATATCATGATAGCGTCACACCTGTTGGCGTATTTTTTCACCGAGTTAAATCACGACCAGGTTCAGAAGGTGGACCAATATCTATATCATATGCGTCTTTCCGACGAGACCCTGTTGGAAATTTCCAAGAGGTTTAGGAAGGAGATGGAAAAAGGTCTAGGCGCAACCACACATCCGACCGCAGCTGTGAAAATGTTGCCCACATTTGTGAGGTCAACACCTGACGGGACTGAACACGGTGAGTTTTTGGCGCTTGATTTAGGGGGAACGAATTTCAGAGTATTGTGGGTAAAGGTTACGGACAATGGACTACAGAAAGTAGAAATGGAGAACCAAATATACGCGATCCCAGAGGATATTATGAGGGGTTCAGGCACTCAACTGTTTGACCATATTGCTGAGTGTCTAGCTAATTTTATGGACAAACTGCAGATTAAAGATAAAAAGTTACCGCTTGGATTTACTTTTTCTTTTCCGTGCCACCAGACGAAACTAGATGAAAGCTTCCTAGTGAGTTGGACGAAAGGCTTCAAATCTTCAGGTGTTGAAGGGAGGGATGTGGTGGCACTTATACGTAAGGCCATACAGAGAAGGGGAGACTTTGATATAGATATAGTCGCGGTAGTAAACGACACCGTTGGCACAATGATGACGTGCGGTTACGATGATCATAATTGTGAAATAGGACTGATTGTTGGTACAGGTAGCAACGCGTGCTATATGGAAGAAATGCGTCACATCGACATGGTAGAGGGAGACGAAGGTAGAATGTGTATTAATATGGAATGGGGGGCATTCGGAGACGACGGATCATTGAATGACATCAGAACGGAGTTTGATCAAGAAATTGACATGGGGTCATTGAATCCGGGAAAACAGTTGTTTGAGAAAATGATTAGCGGAATGTATATGGGAGAGCTGGTTAGGCTGATTTTGGTGAAGATGGCGAAAGAAGAGCTACTATTTGGCGGCAAACTTTCCCCTGAGCTTTTGAACACAGGACGTTTTGAGACAAAAGATATTTCAGACATTGAGGGTGAGAAGGACGGTATACGTAAAGCCAGGGAAGTCCTAATGAGGTTAGGACTAGACCCGACCCAGGAAGATTGTGTGGCGACTCACAGGATTTGTCAAATTGTGTCTACCAGGTCAGCTTCCTTGTGCGCTGCCACCTTAGCCGCCGTGCTACAGAGAATTAAAGAGAATAAGGGGGAAGAGAGGCTGAGATCAACGATTGGCGTAGATGGCAGCGTTTATAAAAAACATCCTCATTTTGCCAAAAGGCTACACAAAACTGTTCGTAGGCTGGTGCCCGGTTGTGACGTTAGGTTCCTGAGGAGCGAGGATGGAAGTGGGAAGGGGGCCGCAATGGTCACCGCCGTCGCTTATAGACTGGCCGACCAGCATAGGGCCAGGCAGAAAACCTTAGAGCACCTACAGCTTTCACATGATCAACTACTAGAAGTCAAGAGAAGGATGAAGGTGGAGATGGAACGTGGACTTTCAAAGGAGACCCATGCGAGTGCACCTGTGAAAATGCTGCCGACTTACGTTTGTGCAACGCCCGATGGAACAGAAAAAGGAGATTTCTTAGCACTTGACCTGGGCGGCACCAATTTCAGGGTGCTGCTGGTTCGTGTAAGAAACGGGAAGTGGGGTGGAGTCGAAATGCATAATAAGATTTATGCCATACCACAAGAGGTGATGCATGGAACAGGGGATGAGTTGTTCGATCATATAGTACAATGCATTGCTGACTTTTTAGAGTACATGGGCATGAAGGGGGTTTCACTACCGCTAGGATTCACTTTTTCATTTCCGTGTCAACAAAATAGCCTTGACGAAAGCATCTTATTAAAATGGACTAAAGGTTTCAAGGCTAGCGGTTGCGAGGGAGAAGACGTTGTGACGTTACTGAAGGAGGCTATCCATAGAAGGGAGGAATTCGATCTAGACGTTGTCGCAGTTGTTAATGACACAGTTGGGACTATGATGACTTGTGGGTTCGAAGATCCTCACTGTGAGGTGGGTTTGATCGTCGGTACCGGAAGCAATGCGTGTTACATGGAAGAGATGCGTAATGTGGAGTTGGTTGAGGGTGAAGAGGGGAGGATGTGTGTCAACATGGAGTGGGGGGCTTTTGGTGATAACGGTTGTCTTGACGACTTCAGGACAGAATTCGATGTCGCCGTGGACGAACTTTCTTTAAACCCGGGTAAACAGAGATTCGAGAAAATGATCAGTGGTATGTATCTGGGGGAAATTGTCAGAAACATTCTTATAGATTTCACTAAGCGTGGATTATTGTTCCGTGGGAGAATTTCTGAGAGGCTTAAAACCAGGGGGATTTTCGAAACTAAATTTCTAAGTCAAATTGAGTCCGACTGTCTTGCGTTATTACAAGTCAGGGCAATCCTACAGCACTTGGGCCTGGAGTCAACATGTGATGATAGTATCATAGTAAAAGAGGTCTGCACCGTGGTAGCTAGGAGAGCGGCACAGTTATGTGGAGCGGGGATGGCAGCAGTCGTTGACAGGATCAGGGAAAATAGGGGGTTGGACGCCTTAAAGGTTACCGTCGGCGTTGATGGAACATTGTATAAACTGCATCCACATTTCGCGAAAGTTATGCACGAGACTGTGAAAGATCTGGCTCCTAAGTGCGATGTTTCCTTTTTACAGTCAGAAGATGGGAGTGGGAAGGGAGCCGCATTAATTACGGCTGTCGCATGTCGTATAAGGGAAGCTGGCCAGAGATACCCATACGATGTTCCTGACTATGCGGGCTATCCGTATGACGTCCCGGACTATGCAGGATCCTATCCATATGACGTTCCAGATTACGCTTGACTCGAGAAA

**Supplemental Fig 1. Effect of *hxk1Δ and glk1Δ* on loss of silencing at the HML locus.** (A) Typical fluorescence images of colonies derived from WT (cyc669), *hxk1Δ* (sk460), *glk1Δ* (sk461) and *hxk1Δ glk1Δ* (sk462) yeast strains containing the LoxP-RFP-LoxP-GFP cassette at URA3 locus and a HMLα::cre at the silenced HML locus. The CRASH (Cre-reported altered states of heterochromatin) system(1) was used in this assay. Bright green sectors in GFP channels or dark sectors in RFP channels represent loss of silencing. (B) The WT, *hxk1Δ, glk1Δ* and *hxk1Δ glk1Δ* yeast strains showed no difference on loss of silence frequency at the HML locus when analyzed by the CRASH (Cre-reported altered states of heterochromatin) assay. Error bar = standard error. ns p>0.05 by Student’s t-test.


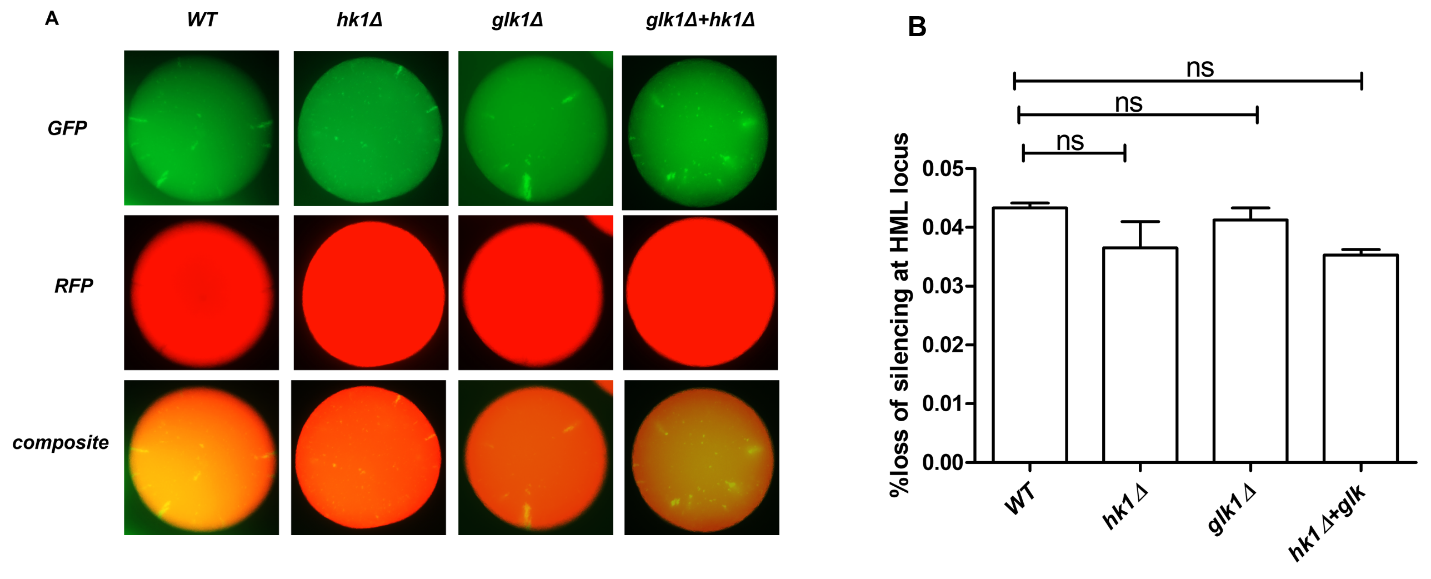


**Supplemental Fig 2. Impact of 2-DG on histone marks level of yeast strains.** (A) Total levels of histone modifications (H3K36me3, H3K27Ac, H3K4me3, H3K56Ac and H4K5,8,12Ac) in the yeast strains treated with 2-DG. Log phase cells were treated with 0.2% 2-DG for 2 hour and Western blot analysis as described in materials and methods. (B) Relative levels of histone modifications comparing to WT cells.


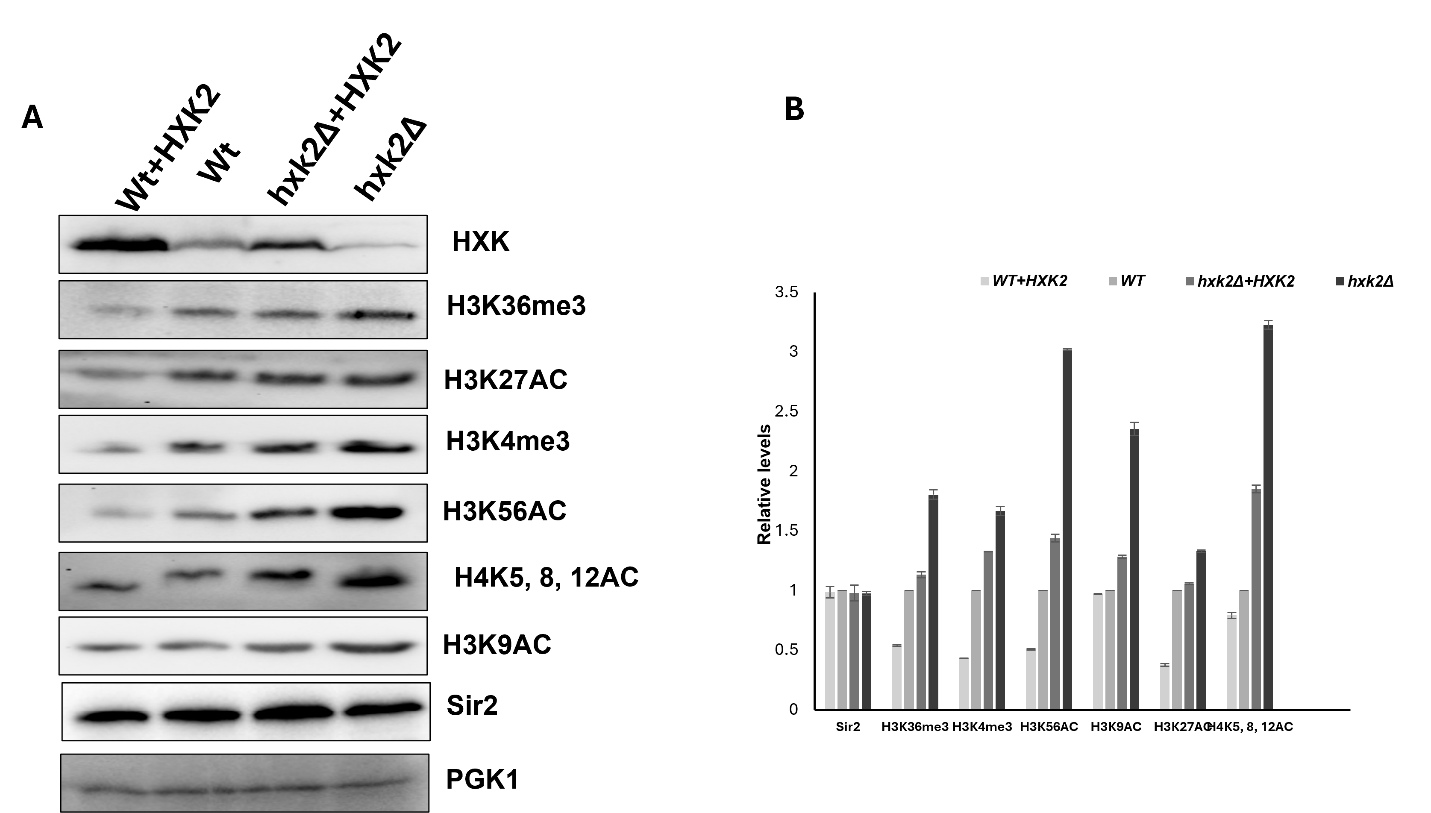


**Supplemental Fig 3. Western analysis of cyclins and γH2A.X.** Western blot analyze the cyclin 2(Cln2), cyclin B1(Clb1) and γH2A.X levels of log-phase yeast from WT+*HXK2*, WT, *hxk2Δ*+*HXK2*, and *hxk2Δ* strains*.*


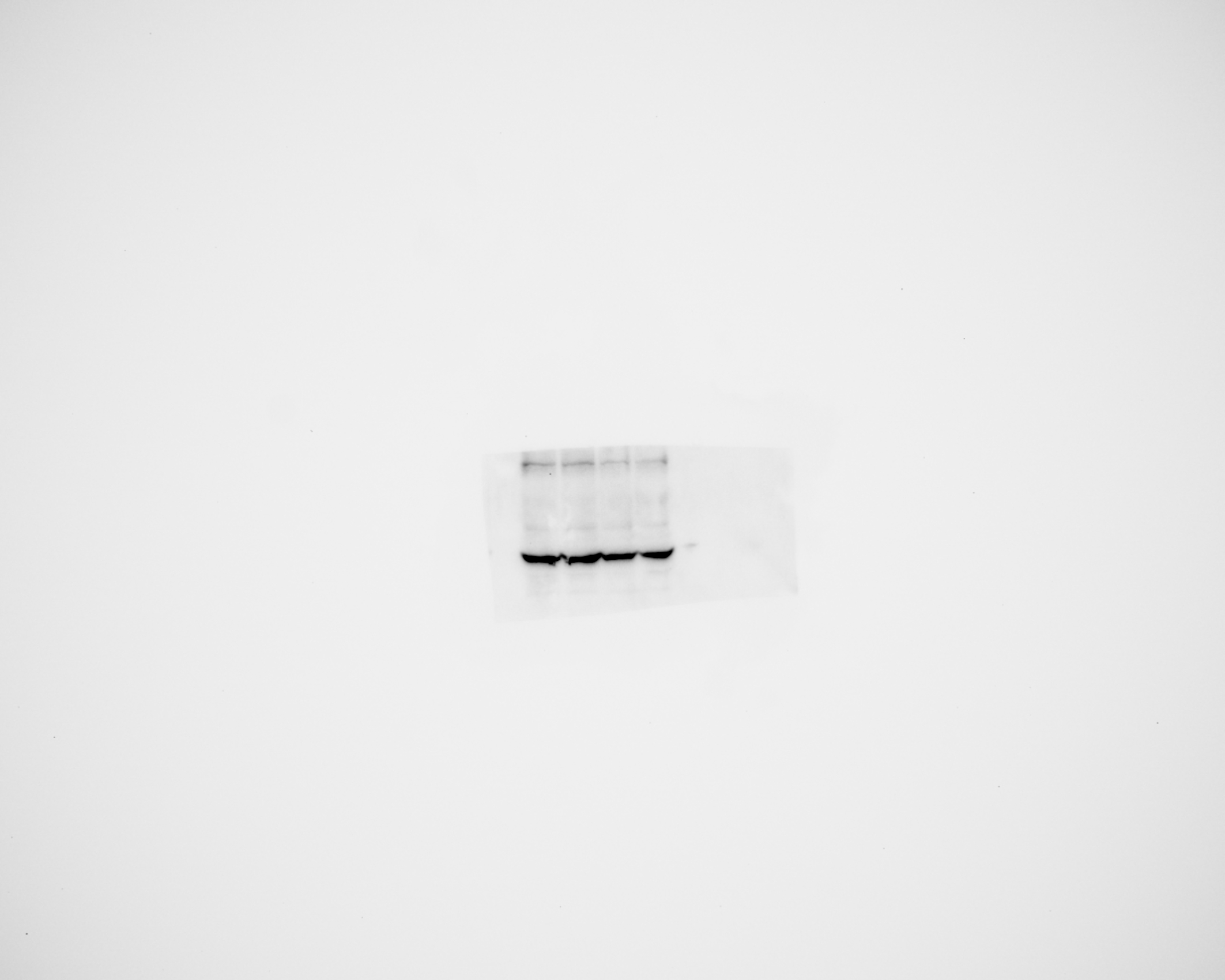

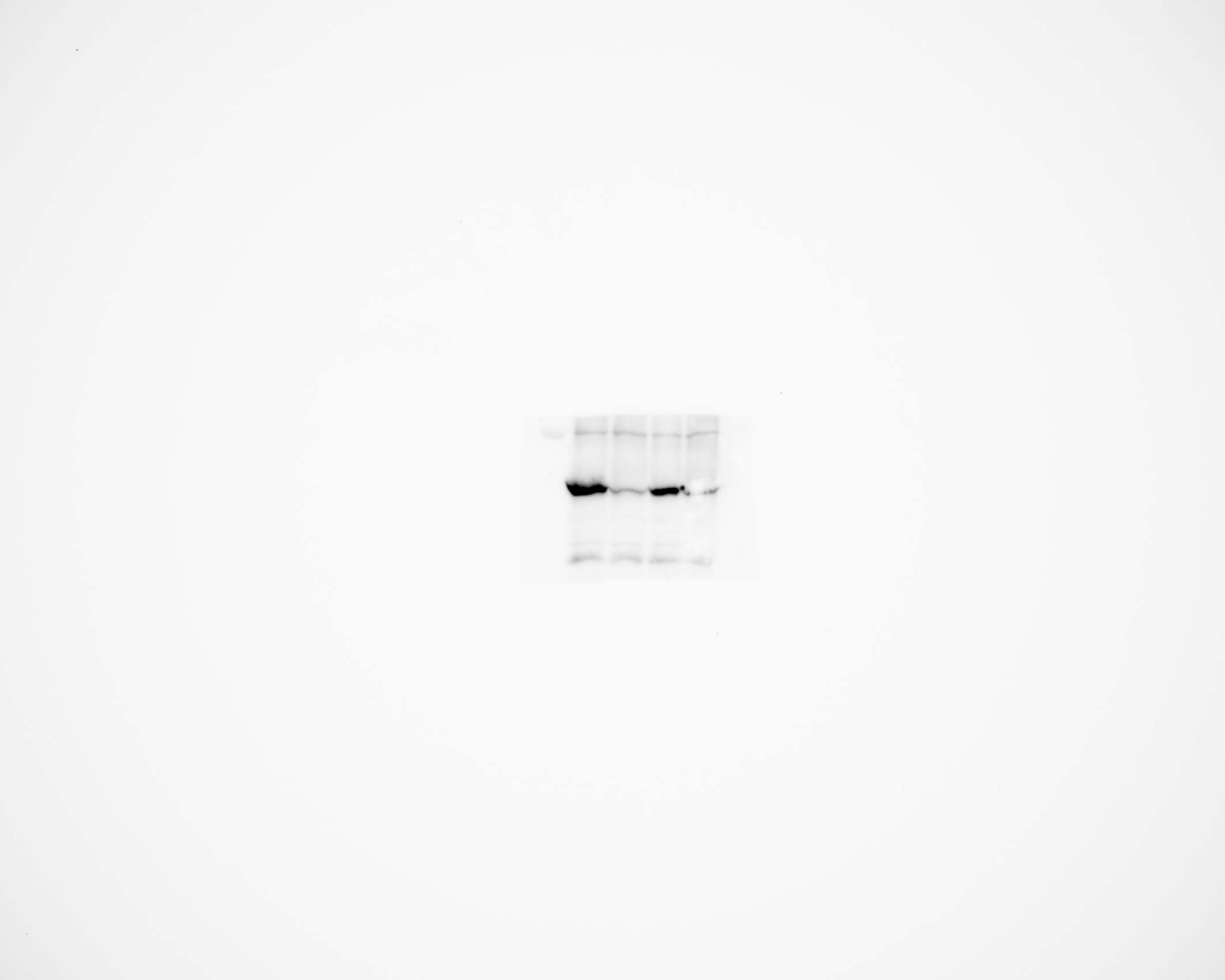

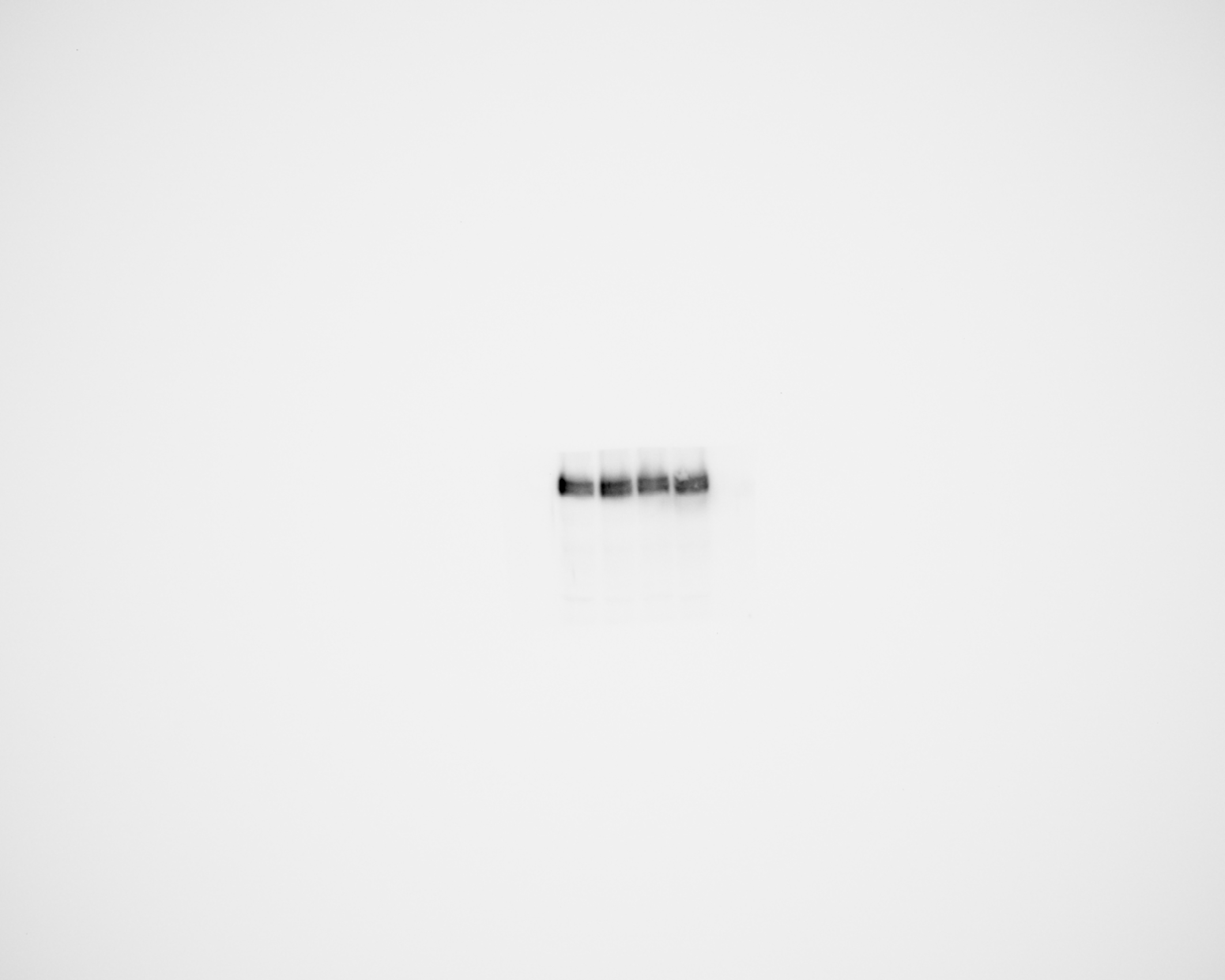

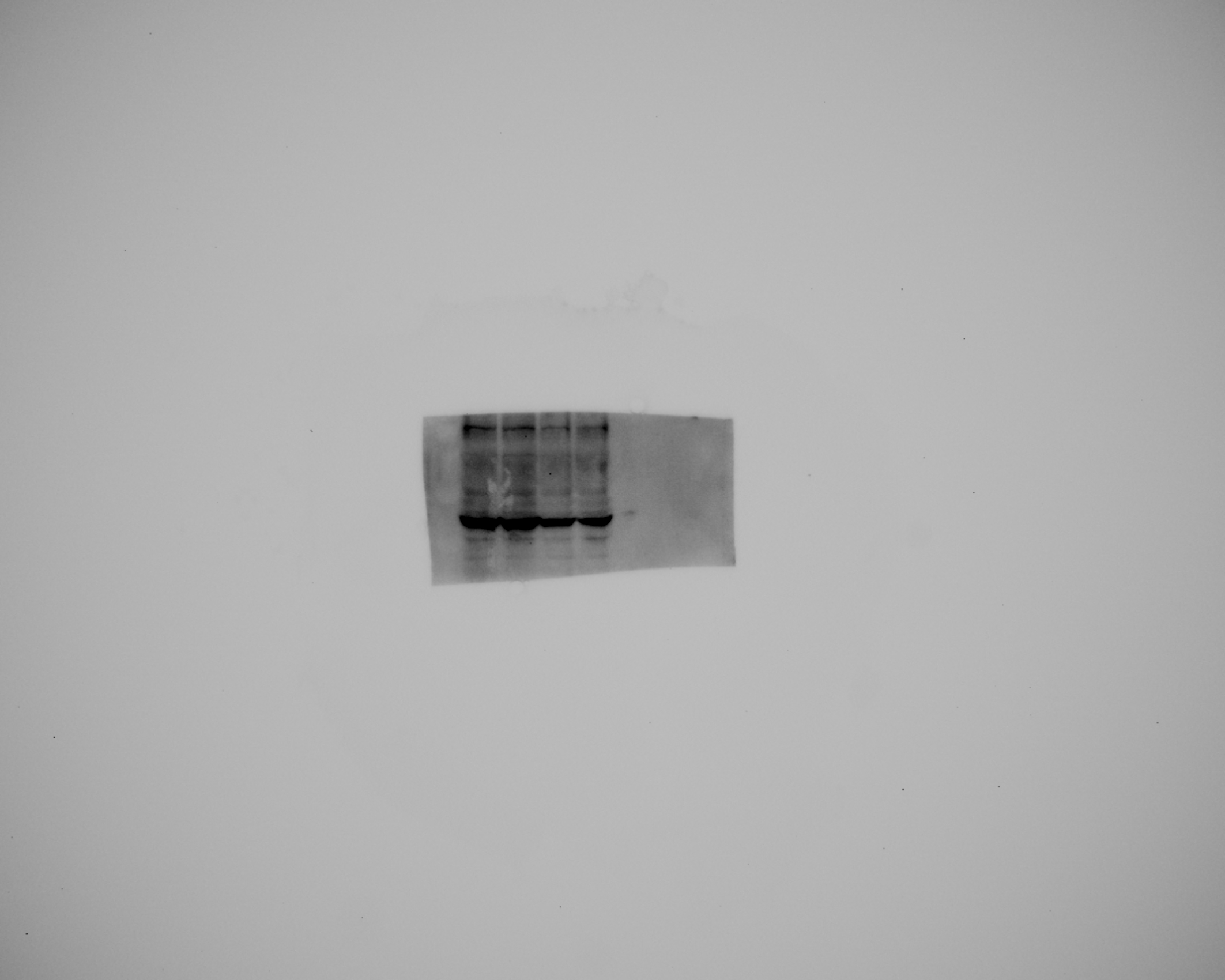


**PGK1**

**HXK**

**Wt+HXK2**

**Wt**

**hxk2Δ+HXK2**

**hxk2Δ**

**CLN2-flag**

**CLB1-flag**


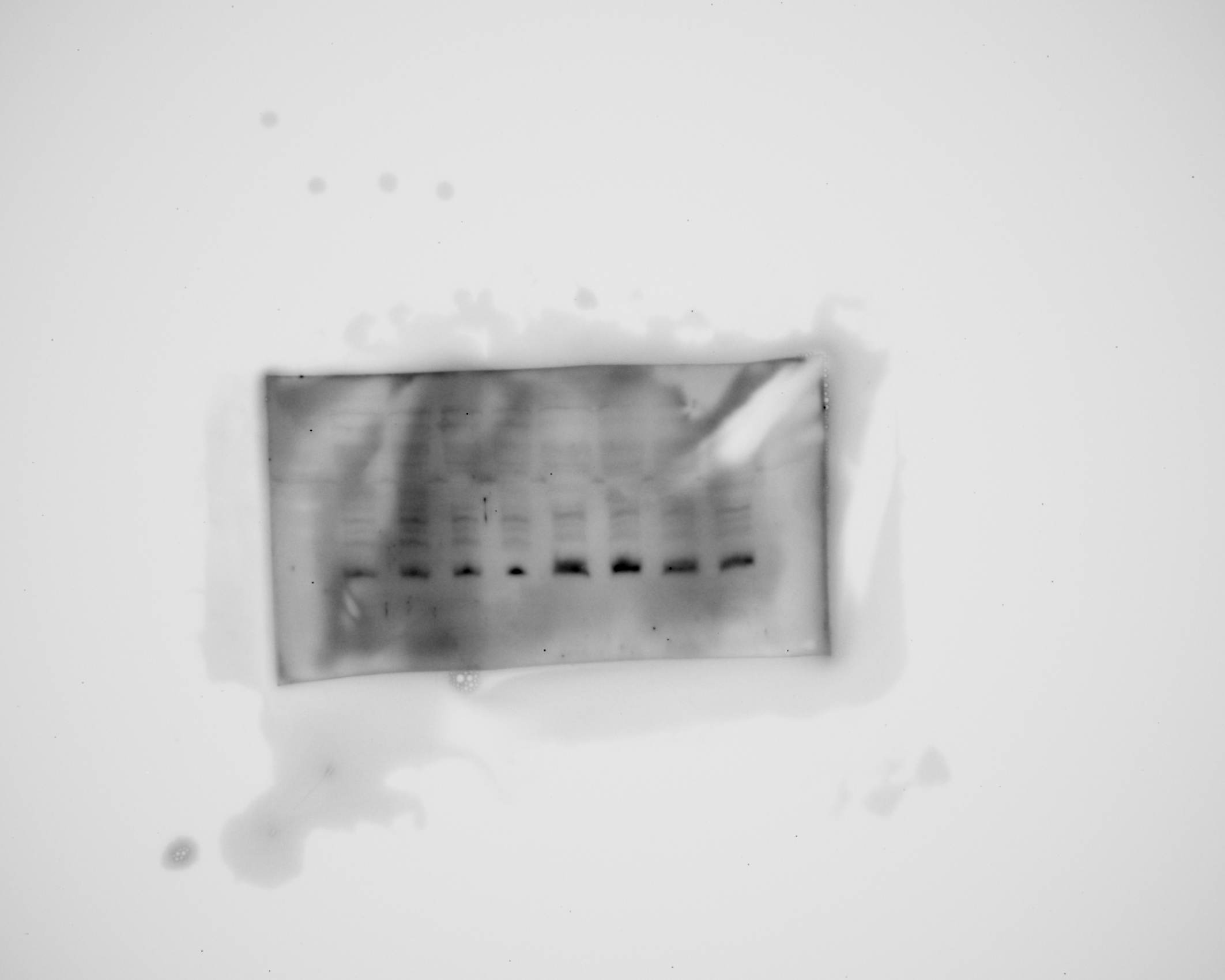


**γH2A.X**

**Supplemental Fig 4. Cellular ATP levels in different strains.** (A) ATP levels in various yeast strains. Log-phase cells were collected, and for the 2-DG treated sample, cells were treated with 0.4% 2-DG for 10 minutes. (B) ATP levels in different yeast strains after 2-DG treatment. Samples were treated with 0.2% 2-DG for 2 hours. Measurement details are provided in the Materials and Methods section. Error bars represent standard error. Error bar = standard error. *p<0.05, **p<0.01, and ***p < 0.001 by Student’s t-test.


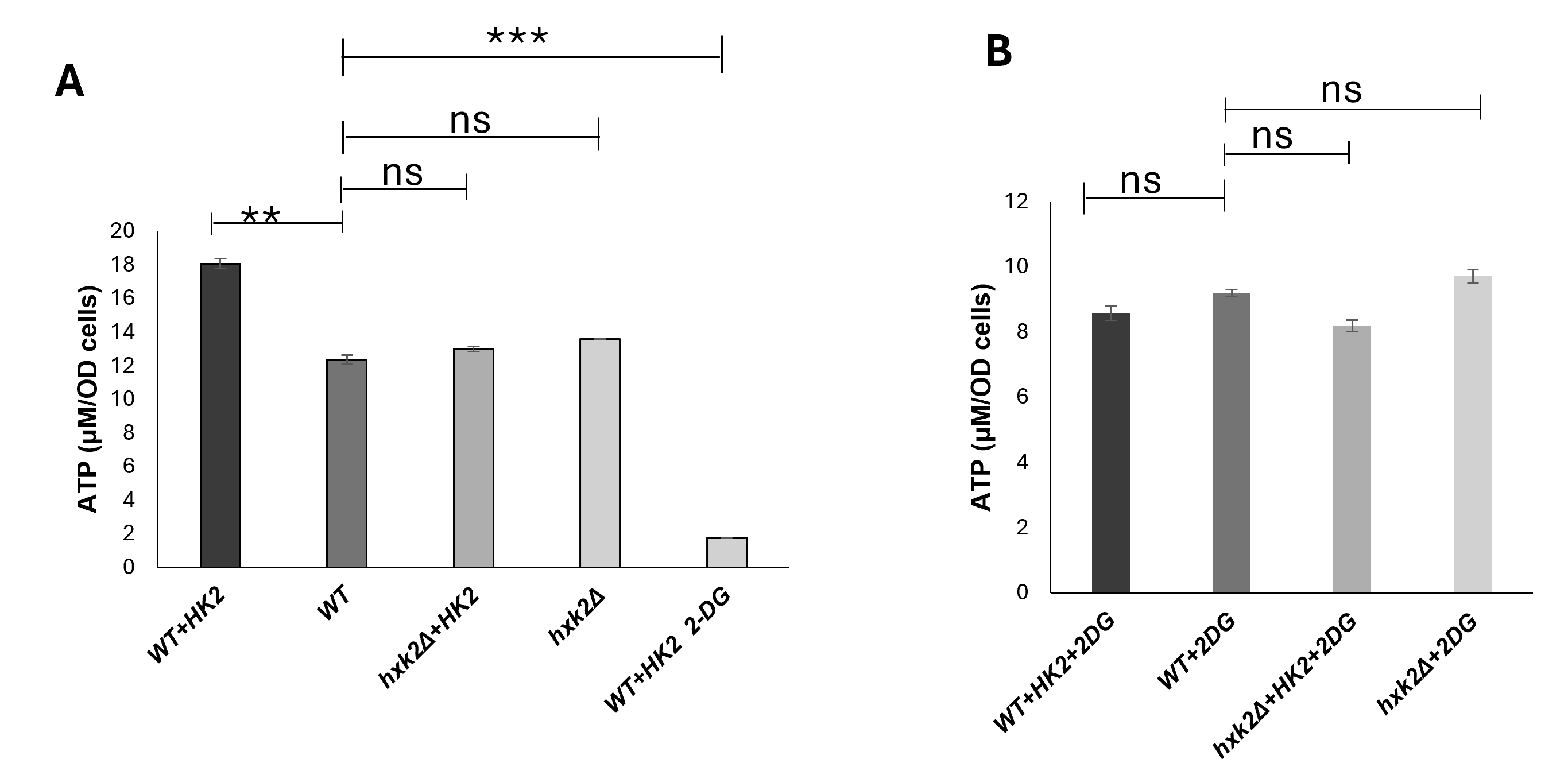


1. Dodson AE, Rine J. Heritable capture of heterochromatin dynamics in Saccharomyces cerevisiae. Elife. 2015;4:e05007.
